# Ectopic Expression of an Atypical Hydrophobic Group 5 LEA Protein from Wild Peanut, *Arachis diogoi* Confers Abiotic Stress Tolerance in Tobacco

**DOI:** 10.1371/journal.pone.0150609

**Published:** 2016-03-03

**Authors:** Akanksha Sharma, Dilip Kumar, Sumit Kumar, Sakshi Rampuria, Attipalli R. Reddy, Pulugurtha Bharadwaja Kirti

**Affiliations:** 1 Department of Plant Sciences, University of Hyderabad, Hyderabad, 500046, India; 2 Department of Postharvest Science of Fresh Produce, ARO, The Volcani Center, Bet Dagan, 50250, Israel; University of Manitoba, CANADA

## Abstract

Late embryogenesis abundant (LEA) proteins are a group of hydrophilic proteins, which accumulate in plants under varied stress conditions like drought, salinity, extreme temperatures and oxidative stress suggesting their role in the protection of plants against these stresses. A transcript derived fragment (TDF) corresponding to *LEA* gene, which got differentially expressed in wild peanut, *Arachis diogoi* against the late leaf spot pathogen, *Phaeoisariopsis personata* was used in this study. We have cloned its full length cDNA by RACE-PCR, which was designated as *AdLEA*. *AdLE*A belongs to the atypical Group 5C of LEA protein family as confirmed by sequence analysis. Group 5C LEA protein subfamily contains Pfam LEA_2 domain and is highly hydrophobic. In native conditions, expression of *AdLEA* was upregulated considerably upon hormonal and abiotic stress treatments emphasizing its role in abiotic stress tolerance. Subcellular localization studies showed that AdLEA protein is distributed in both nucleus and cytosol. Ectopic expression of *AdLEA* in tobacco resulted in enhanced tolerance of plants to dehydration, salinity and oxidative stress with the transgenic plants showing higher chlorophyll content and reduced lipid peroxidation as compared to wild type plants. Overexpressed *AdLEA* tobacco plants maintained better photosynthetic efficiency under drought conditions as demonstrated by chlorophyll fluorescence measurements. These plants showed enhanced transcript accumulation of some stress-responsive genes. Our study also elucidates that ROS levels were significantly reduced in leaves and stomatal guard cells of transgenic plants upon stress treatments. These results suggest that *AdLEA* confers multiple stress tolerance to plants, which make it a potential gene for genetic modification in plants.

## Introduction

Plants being sessile in nature are constantly challenged by adverse environmental conditions. These diverse abiotic stresses including drought, salinity, oxidative and temperature disrupt cellular homeostasis thereby affecting the growth and development of plants. Plants have developed different adaptive mechanisms to cope up with these challenges and minimize the damage. One such mechanism is the expression of osmotically active late embryogenesis abundant (LEA) proteins during desiccation or osmotic stresses associated with low temperature [1]. LEA proteins are a group of highly hydrophilic proteins, which were first identified in cotton as proteins that accumulate during late maturation stage of seed development [2]. Since then, many LEA proteins have been identified and characterized in plants ranging from algae [3], moss [4], ferns [5], angiosperms [6] and even in anhydrobiotic arthropods [7] and bacteria [8] in response to water limiting conditions. The expression of LEA proteins is not only restricted to embryonic tissues, but also to vegetative tissues of plant in water deficit conditions during various environmental and physiological stresses. Their expression can also be induced in response to exogenous application of abscisic acid, drought, high salinity, sub-optimal temperatures and osmotic conditions on plants implying their role in abiotic stress tolerance mechanism in plants [9]. Functional insights of LEA proteins during desiccation tolerance reveal their role in the transportation of nuclear-targeted proteins during stress [10], stabilizing membrane structures, as antioxidants by binding to metal ions, increasing cellular mechanical strength by the generation of filaments [11] and protection of cell membranes against desiccation [12]. They are also known to prevent protein aggregation and enzyme inactivation at low temperature [13], heat and desiccation stress [14]. They are also proposed to contribute to the formation of tight glass matrices together with sugars in desiccated cells [15]. Sub-cellular localization studies of LEA proteins revealed that they are not transmembrane proteins. Rather, they have been found to be localized in almost all cellular compartments including the chloroplast [16], mitochondria [17], cytoplasm [18], nucleus [19], endoplasmic reticulum [20], vacuole [21] and even in the vicinity of plasma membrane [22] where they are proposed to exert their protective function acting as chaperones to repair improperly folded proteins [23].

LEA proteins are variable in size ranging from 5 to 77 kDa and belong to a multigene protein family [24]. One unifying and outstanding feature of LEA proteins with the exception of group 5 is that they are highly hydrophilic proteins rich in amino acids like glycine, glutamate and/or alanine, serine and threonine and majority of them lack or contain fewer tryptophan and cysteine residues [25]. Most LEA proteins exist primarily as randomly coiled, intrinsically disordered proteins in solution. This flexible conformation of LEA proteins helps them in binding to macromolecules and protecting enzymes [26]. The traditional criterion to classify the LEA proteins was based on the sequence homology of amino acids and conserved motifs from different plant species [27]. However, with the discovery of more LEA proteins and development of computational analysis methods, different criteria to classify LEA proteins were demonstrated. Presently, the nomenclature of LEA proteins is different in different classification methods [27];[28];[29];[30]. According to classification introduced by Battaglia’s group, LEA proteins are classified into seven distinct families. Groups 1, 2, 3, 4, 6 and, 7 are hydrophilic and have specific motifs within each group and hence, are considered typical or genuine LEA proteins. Since group 5 lacks a significant motif or consensus sequence with a significantly higher proportion of hydrophobic amino acids, it is considered an atypical LEA protein [29]. Group 5 LEA proteins are non-homologous proteins, which on the basis of sequence similarity are further divided into 3 subgroups corresponding to the first described proteins in group, 5A (D-34), 5B (D-73) and 5C (D-95) [31];[29]. According to Pfam database, this subgroup is classified as 5A (SMP), 5B (LEA_3) and 5C (LEA_2) with corresponding Pfam No’s PF04927, PF03242, and PF03168 respectively. A distinct property of group 5 members is that they are insoluble after boiling suggesting that they are probably heat labile and may adopt a globular conformation upon heating [32];[33]. Moreover, among group 5, group 5C LEA proteins have some distinct properties, which make them unique among the LEA protein family. These proteins are natively folded and contain a larger proportion of β- sheets than α- helices in the hydrated state as opposed to other LEA family proteins, which are intrinsically unstructured [33]. Presently, LEA14-A from *Arabidopsis*, which is a group 5C protein is the only member from LEA family, which was confirmed to have a defined secondary and tertiary structure in solution [32]. Other characteristic properties of group 5C LEA proteins include lower instability index, narrow range of GRAVY values and a low proportion of polar (hydrophilic) and smaller amino acids but with a high proportion of non-polar (hydrophobic) amino acids [34]. All these differences imply that Group 5C LEA proteins might function differently from other groups of LEA proteins.

Since the discovery of first LEA proteins in cotton, the emphasis has been centered on elucidating the role and function of these proteins in plants. Most attempts to understand their functions had been aimed at characterizing and overexpressing the concerned novel gene in different organisms, be it plant species, yeast or bacteria. Presently, only a small fraction of group 5C LEA proteins have been cloned and characterized. Among them many proteins have been reported to be associated with resistance to multiple stresses [34];[35];[36]. Overexpression of maize group 5 LEA gene Rab28 resulted in enhanced water stress tolerance of transgenic maize plants [37]. JcLEA, a group 5, LEA-Like protein from *Jatropha curcas* conferred a high level of tolerance to dehydration and salinity when overexpressed in *Arabidopsis thaliana* [6]. Also, SiLEA14, an atypical group 5C LEA protein conferred salt and osmotic stress to foxtail millet [38]. In addition a Group 5 LEA protein from *Zea mays*, ZmLEA5C enhanced tolerance to osmotic and low-temperature stresses when expressed in tobacco and yeast [39] and recently *RcLEA*, a late embryogenesis abundant protein gene isolated from *Rosa chinensis* was reported to, confer tolerance to *Escherichia coli* and *Arabidopsis thaliana* under various abiotic stresses [40].

Wild relatives of many plant species particularly crops like wheat, maize, potato, tomato, cotton, tobacco and sugar cane [41];[42] are extremely important as they contain high levels of resistance to several important biotic and abiotic stresses. Discovery and incorporation of resistant genes from these wild species to less resistant or susceptible species hold a great potential for genetic enhancement and sustaining crop improvement and productivity. *Arachis diogoi* Hoehne (Syn. *A*. *chacoense*) is a diploid wild relative of cultivated peanut *Arachis hypogaea* L. There are many reports that confirm that *A*. *diogoi* is highly resistant to Late Leaf Spot (LLS) causing pathogen, *Phaeoisariopsis personata* as well as several other fungal and viral pathogens [42];[43]. In the previous study from our group, *Arachis diogoi* was challenged with late leaf spot pathogen, *Phaeoisariopsis personata*, and a transcript derived fragment (TDF) corresponding to a LEA gene was identified using differential gene expression study [44]. In the present study, the full-length cDNA sequence was cloned from this partial fragment by RACE-PCR and the gene was designated as *AdLEA*, and the deduced protein belongs to group 5C family of LEA proteins on the basis of sequence similarity. Expression studies of *AdLEA* were done in native conditions in response to different treatments and its subcellular localization was done employing both N and C-terminal translational GFP fusions. For functional characterization of *AdLEA* against multiple abiotic stresses, it was overexpressed in tobacco and the transgenic plants were analyzed for drought, salinity, and oxidative stresses. These results are discussed in the present communication to gain further insights into the function of this unique group 5C member of LEA protein

## Materials and Methods

### 5'/3' RACE-PCR and Isolation of Full-Length cDNA of *AdLEA*

RACE-PCR was performed to obtain the full length sequence of *AdLEA* gene using SMART^TM^ RACE cDNA Amplification kit (Clontech, USA) following manufacturer’s instructions. The 5ʹ and 3ʹ RACE products using 5'AdLEA-GSP1 and 3'AdLEA-GSP1 primers respectively were aligned with the partial sequence of *AdLEA* obtained from preliminary differential gene expression study using cDNA-AFLP in *A*. *diogoi* after infection with *P*. *personata* [44] to obtain full-length sequence of *AdLEA*. Finally, the open reading frame of *AdLEA* was amplified using Phusion^®^ High-Fidelity DNA polymerase (Finnzymes, New England BioLabs, Ipswich, MA) with primers having specific restriction enzyme sites for cloning in pRT100 vector. Primers used in this step are AdLEA-F and AdLEA-R flanked by restriction sites for *Sac*1 and *Kpn*1 respectively. All the primer sequences mentioned and otherwise used in the study are listed in (Table A in S1 File).

### cDNA Sequence and Protein Analysis

Sequence analysis was performed using BLASTx and BLASTp [45]. Nucleotide translation, prediction of theoretical molecular mass and the isoelectric point of AdLEA protein were performed using Expasy tool (http://expasy.org/tools/pi_tool.html) [46]. SignalP 4.1 was used for predicting the signal peptide within the protein [47]. Motif analysis was performed using the Pfam program (http://www.ebi.ac.uk/tools/InterProScan). Multiple sequence alignment was performed by ClustalW [48] and phylogenetic tree construction was done by MEGA 5.1 software [49]. The grand value of hydropathy (GRAVY) analysis and instability index for deduced amino acid sequence were predicted with PROTParam (http://au.expasy.org/tools/protparam.html) and PSORT (http://psort.nibb.ac.jp) programs [50]. Analysis of protein hydropathy was done by constructing hydropathy plot with Kyte and Doolittle algorithm (http://ipsort.hgc.jp/) [51]. Subcellular localization predictions were performed using PSORT II (http://psort.hgc.jp/form2.html) [52]; MultiLOC (http://abi.inf.uni-tuebingen.de/Services/Multiloc) [53]; and Y LOC (http://abi.inf.uni-tuebingen.de/Services/YLOC/webloc.cgi) [54].

### Plant Material and Stress Treatments in Gene Expression Studies of *AdLEA* in *A*. *diogoi*

Tobacco (*Nicotiana tabacum* var. Samsun), wild peanut (*A*. *diogoi*) accession number ICG-8962 (kindly provided by ICRISAT, Patancheru, India) and *Nicotiana benthamiana* plants were used in this study and maintained in separate greenhouses at 24±1°C with a photoperiod of 14/10 h of light /dark with light intensity of 100 μmol m^-2^s^-1^. Stress and hormonal treatments on *Arachis* plants were done according to protocol standardized by Kumar et al., 2011 [55]. In brief, twigs from two-month-old plants of *A*. *diogoi* were cut with a sharp sterilized blade, washed with sterile distilled water and kept in trays lined with moist filter paper. Cut ends of shoots were wrapped properly with water soaked cotton and whole set up was sealed with polythene covers to maintain moist condition. This helps the shoots to get acclimatized in a growth room at 25±1°C (with a photoperiod of 16 h of light and 8 h of dark, light intensity of 60 μmol m^-2^s^-1^) and subsequently enhance adventitious roots formation at the cut ends of shoots. These shoots with adventitious roots were subjected to different hormonal treatments namely 100μM salicylic acid (SA), 100μM methyl jasmonate (MeJA), 100μM abscisic acid (ABA), 250μM ethephon and 100μM sodium nitroprusside (SNP). The trifoliate compound leaves of *Arachis* were used as a sample in each stress, sufficient enough to isolate 2 μg of RNA (taken in duplicates). This constitutes one biological sample for the experiment. Heat (high-temperature stress) and cold (low-temperature stress) treatments to leaf samples were applied by keeping them at 42°C and 4°C respectively. For other stress treatments, samples were treated with 200 mM sorbitol, 100 mM NaCl, 10% (w/v) polyethylene glycol (PEG-MW6000) and 10 μM methyl viologen (MV). Samples were collected in liquid nitrogen at regular intervals of 0 min, 5 min, 15 min, 30 min, 1 h and 2 h in case of heat, and 0 h, 3 h, 6 h, 12 h and 24 h in rest of the treatments. The samples were stored at –80°C for experimental studies.

### Gene Expression Profile of *AdLEA* in Native Conditions

Total RNA was extracted from leaf samples (subjected to various treatments) according to Chang et al., [56]. Two microgram of total RNA was used for first-strand cDNA synthesis by an oligo dT (18 mer) primer using SMART MMLV Reverse Transcriptase (Clontech, Becton Dickinson, USA). Diluted first strand cDNA samples were subjected to qRT-PCR with gene-specific primers for *AdLEA* (Table A in S1 File) and 10 μl SYBR^®^ Premix Ex Taq with ROX (Takara Bio Inc., Japan). Three independent biological replicates with three technical replicates of each biological replicate for each sample were used for analysis including three non-templates, which served as negative control (non-template control with just water without cDNA). qRT-PCR was carried out in Realplex (Eppendorf, Germany) amplifier with the following program: 95°C for 5 min; 40 cycles of 95°C for 15 s, 58°C for 20 s, 72°C for 30 s followed by the melting curve to ensure a single product of each amplicon. Alcohol dehydrogenase class III (*adh3*) and polyubiquitin (*UBI1*) were used as internal controls to calculate relative quantification of gene expression [57]. The threshold cycle (C_T_) was used to calculate relative fold change (RFC) in RNA expression at each time point of the treated sample compared to control conditions by using the formula, fold change = 2^-(ΔCt treated-ΔCt control)^ [58].

### Sub-Cellular Localization Studies of AdLEA Protein

GFP was fused translationally with *AdLEA* at both N-terminal (GFP: *AdLEA*) and C-terminal (*AdLEA*: GFP) in pEGAD and pCAMBIA1302 vectors respectively for AdLEA localization studies [59]. *AdLEA* Open Reading Frame (ORF) was amplified with primers AdLEA-pEGAD-F and AdLEA-pEGAD-R having restriction sites for *Eco*RI and *Hin*dIII respectively, (Table A in S1 File) and cloned into pEGAD vector after digestion with above-mentioned restriction enzymes, for generating the N-terminal fusion with GFP. Similarly, to generate C-terminal GFP fusion, stop -codon- less, coding region of *AdLEA* was amplified with primers AdLEA-pC1302-F and AdLEA-pC1302-R having restriction sites for *Bgl*II and *Spe*I respectively (Table A in S1 File) and cloned into pCAMBIA1302 vector after digestion with the same set of restriction enzymes. Both fusions were confirmed by sequencing. The recombinant vectors and empty pCAMBIA1302 vector generating free GFP serving as control were mobilized into *Agrobacterium* strain LBA4404 by the freeze-thaw method. Agroinfiltration and microscopic studies were performed according to Yang *et al*. [60]. The overnight-grown cultures of *Agrobacterium* with both constructs and control vector were pelleted down and resuspended in infiltration medium (10mM MES, 10mM MgCl_2,_ and 200μM acetosyringone) and infiltrated into the adaxial side of *N*. *benthamiana* leaves after adjusting the OD value to 0.6. Leaves were visualized for GFP expression 72–96 hours post infiltration (hpi) at different time points with laser scanning confocal microscopy (Leica TCS SP2 with AOBS, Heidelberg, GmbH, Germany) with excitation and emission wavelengths at 475–495 nm and 520–560 nm respectively. The experiment was performed in triplicates. Protein extraction and subsequent immunoblot analysis were done to check the expression and stability of AdLEA: GFP fusion protein in cells following the protocol as described by Kumar and Kirti [61]. Western Blot analyses was done using, anti-mouse Histidine primary antibody, as GFP was tagged with Histidine in pCAMBIA1302 vector, which was detected with ALP-conjugated goat anti-rabbit secondary antibody using BCIP/NBT as substrate.

### Construction of Recombinant *AdLEA* Binary Vector and Genetic Transformation of Tobacco

The *AdLEA* ORF was amplified with primers AdLEA-F and AdLEA-R having restriction sites for *Sac*I and *Kpn*I respectively (Table A in S1 File) and cloned into corresponding sites of pRT100 vector. The expression cassette that has *AdLEA* flanked by CaMV35S promoter and polyadenylation signal was excised with *Hin*dIII and subsequently sub-cloned into binary vector pCAMBIA2300 at the same site. The recombinant vector pCAMBIA2300-*AdLEA* having kanamycin as marker gene was transformed into *Agrobacterium* strain LBA4404 by freeze-thaw method and used for transformation of tobacco plants (*N*. *tabacum* cv. Samsun) by leaf disc method according to Horsch et al. [62]. The transformants were selected on 125 mg L^-1^ kanamycin. Genomic DNA and total RNA were isolated from T_0_ *AdLEA* transformants for molecular analysis. RNA was reverse transcribed and PCR with specific primers for the target gene as well marker gene *nptII* was undertaken. High and low expression plants were identified and their progenies were maintained for further analysis. All experimental analyses were conducted in T_2_ generation of transgenic plants.

### Chlorophyll Fluorescence Measurements of *AdLEA* Transgenic Plants Using Pulse-Amplitude-Modulation (PAM) Chlorophyll Fluorometer after Drought Treatment

Chlorophyll fluorescence measurements were performed in drought-induced two-months-old *AdLEA* transgenic plants to check for the photochemical efficiency under drought stress in greenhouse conditions. Three-week-old seedlings of wild-type (WT) and three high expression transgenic lines (#2, 4 and 9) grown on half strength MS medium without organic nutrients were transferred to soil in plastic cups for further growth of two weeks. WT and transgenic plants of same age and size were then shifted individually to separate pots in the greenhouse and allowed to grow for ten days. After acclimatization, these plants were subjected to drought stress by withholding water for 18 days, after which significant differences in wilting were observed. Plants were watered on the 18^th^ day and kept for recovery for three days. Chlorophyll fluorescence measurements were performed on leaves at the third to fourth position from shoot apex of drought treated WT and transgenic plants with MINI-PAM (Walz, Effeltrich, Germany). The measurements were taken on D0, D6, D12, and D18 (D–day) after drought treatment followed by a recovery (R) period of three days for both WT and transgenic lines. The leaves were first dark adapted for 30 min by fixing leaf clips to ensure that all photosystem-II (PSII) reaction centers (RCs) were open. The potential maximum quantum yield (*F*_*v*_*/F*_*m*_) was recorded by illuminating the leaves with a beam of saturating light (intensity– 4500 μmol m^-2^ s^-1^ of 650 nm peak wavelength, an excitation intensity sufficient to ensure closure of all PS-II RCs) focused on the leaf surface using a special leaf clip holder (model 2030-B, Walz) described by Bilger et al. [63]. This leaf clip holder allows the measurement of light and saturating light pulses that fall on a leaf at an angle of 60°. Further, steady-state fluorescence (F_s_) measurement was performed after continuous illumination with white actinic light and a second saturating pulse (4500 μmol m^-2^ s^-1^) was imposed to determine the effective photochemical quantum yield F′/F_m_′, also known as [Y(II)] in the light-adapted state.

### Abiotic Stress Tolerance Assays for Transgenic *AdLEA* Plants

Stress tolerance assays were performed at the young seedling stage and on mature plants by leaf disc assays following the protocols [64];[65]. We selected two high expression transgenic lines (#2 and 4) and one low expression transgenic line (#7) for different stress tolerance assays. The experiments were performed on the homozygous T_2_ generation of transgenic plants and repeated three times. Seeds from all three transgenic lines and WT plants were surface-sterilized by 2% (v/v) sodium hypochlorite solution for 10 min by continuous shaking, rinsed 4–5 times with sterile distilled water and were allowed to germinate on half strength MS medium with 125 mg L^-1^ kanamycin (for transgenic) and without kanamycin (for WT), devoid of organic nutrients. Seedlings were maintained in culture room at 25±1°C with a photoperiod of 16/8 h light/dark with light intensity 60 μmol m^-2^s^-1^ for 11 days and then shifted to corresponding stress medium plates having half strength MS (MSH) without organic nutrients, i.e. 200mM and 300mM NaCl for salt stress, 10% and 12% (w/v) PEG (corresponding to final osmotic potential of—0.123 and -0.184 MPa) [66], for dehydration stress and 200 mM and 300 mM sorbitol for osmotic stress. Duration of stress varied for different stress treatments, depending upon the visible phenotypic changes observed between the seedlings of WT and transgenic lines. Oxidative stress was applied by placing the seedlings on MSH medium containing 5 and 10 μM MV for 4 d and then shifted to the stress-free recovery medium. [11 seedlings per plate were kept (in duplicate) and together they made one biological set of experiment. Likewise, three experiments were done].

For leaf disc assays leaf discs of 1 cm radius were punched from healthy, fully distended leaf of two months old transgenics as well as WT plants with the help of cork borer. [15 leaf discs from a single leaf were used as a sample for stress treatment (in duplicate). This constitutes one biological sample. Similar experiments were repeated three times]. Discs were floated on 10 mL solution of 0, 200 and 300mM NaCl for salt stress, 0, 10, 12 and 14% PEG (corresponding to final osmotic potential of -0.123, -0.184, -0.256 and -0.341) for dehydration stress, 0, 300, 400 and 500mM sorbitol for osmotic stress and 0, 5 and 10μM MV for oxidative stress treatments respectively. The treatments were carried out in continuous white light at 25± 1°C until visible differences were observed among the lines.

### Biochemical Analysis—Total Chlorophyll Content, Lipid Peroxidation and Proline Estimation

Total chlorophyll content was estimated spectrophotometrically after extraction of fresh material in 80% acetone according to Arnon [67]. [for seedling assays—200 mg, for disc senescence assay and drought stress under field conditions—500 mg of fresh weights was taken]. The extent of lipid peroxidation was determined by measuring the thiobarbituric acid reactive substances (TBARS) [68]. Leaf discs (500mg) after different stress treatments were extracted in a solution of 0.5% w/v TBA in 20% trichloroacetic acid and the absorbance was measured at 532 and 600 nm. The malondialdehyde (MDA) levels were estimated using the extinction coefficient 155 mM^-1^ cm^-1^. The proline content in leaves was estimated according to Bates et al., [69]. The leaf samples from unstressed (D0) and drought stressed (D18) transgenic and WT plants were collected and frozen in liquid nitrogen. Frozen leaf tissue (100mg) was ground to a fine powder in liquid nitrogen and extracted with 4 ml of 3% sulpho salicylic acid. The homogenate was filtered through filter paper and incubated at 100°C for 10 min. The absorbance of homogenate was measured spectrophotometrically at 520 nm using toluene as blank. The proline concentration was determined as μmole g^-1^ FW.

### Reactive Oxygen Species (ROS) Quantification in *AdLEA* Transgenic Tobacco Using 2',7'-Dichlorodihydrofluorescein Diacetate (H_2_DCFDA) and Nitro Blue Tetrazolium (NBT) Staining

Confocal studies were done to detect total cell ROS using H_2_DCFDA, against dehydration stress induced with PEG [70]. Single leaf from two-months old mature plants was used in one experiment (in duplicates), which was repeated thrice. Briefly, epidermal peels from abaxial leaf surface of transgenic lines and WT plants were treated with H_2_DCFDA for 20 min and kept in the dark. The excess dye was removed by three washes with washing buffer (30mM KCl + 10mM MES-KOH). Stress was induced by incubating peels in 10% (w/v) PEG solution for 30 min. Epidermal peels treated with only buffer solution were kept as controls, to detect the basal levels of ROS in the guard cells of the WT and transgenic plants. The fluorescence was captured by Laser Scanning Confocal Microscope (Leica, TCS-SP2 with AOBS, Heidelberg, GmbH, Germany) with excitation and emission wavelengths at 475–495 nm and 517–527 nm respectively. Fluorescence quantification was done using Image-J 1.42 software (NIH, USA) by selecting appropriate areas of pigmentation. For detection of Superoxide anion (O_2_^-^), histochemical studies using NBT staining was done against salinity stress induced with NaCl according to Driever et al., [71] with minor modifications [72]. Briefly, petioles of one month old transgenic plants and WT were cut under water to avoid obstructions in the vasculature. Two days before the experiment, all plants were watered or treated with 100mM NaCl solution. Petioles were soaked in 6mM NBT dissolved in 10mM potassium phosphate buffer (pH 7.8) for probe feeding of the leaves. Subsequently, the treated leaves were transferred to continuous white light for 15 min to assess the effect of photosynthesis on superoxide production. Colored leaves were then boiled in 4:1:1 (v/v/v) solution of ethanol, lactic acid, and glycerol to remove chlorophyll. Finally, to quantify formazan formation, leaves were boiled in dimethyl sulfoxide until they were clear and formazan concentration was measured spectrophotometrically at 560 nm.

### Expression Analysis of Stress-Related Genes in *AdLEA* Transgenic Tobacco

Relative gene expression studies of some candidate genes expressed in abiotic stress conditions were performed in transgenic lines as compared to WT with or without induced stress. Leaf samples from unstressed (D0) and drought stressed (D18) plants were collected from representative high expression transgenic line 2 and WT plants. Single leaf from each plant constituted one biological sample and the experiment was done in triplicates. Samples were frozen in liquid nitrogen and kept at –80°C until further use. RNA was isolated from frozen samples using RNeasy Plant Mini Kit (Qiagen, Germany) and subjected to quality check and reverse transcription. Two microgram of total RNA was used for first-strand cDNA synthesis and quantitative real-time PCR was performed as described earlier. Gene-specific primers for various stress-related genes, 18S rRNA, and polyubiquitin (*NtUBI1*), which were used as reference genes to calculate relative quantification of gene expression are listed in (Table B in S1 File). C_T_ value was used to calculate RFC by ΔΔC_T_ method [58].

### Statistical Analysis

The data analysis was done by analysis of variance (one-way ANOVA and two-way ANOVA) using GraphPad Prism ver. 5.0 and the mean values were compared by the student Newman-Keuls analysis and Bonferroni post-tests. All the experiments were performed in triplicates. Details of the individual sample size for each analysis is mentioned in the figure legends.

## Results

### Isolation of Full-Length *AdLEA* cDNA and Sequence Analysis of AdLEA Protein

A transcript derived fragment of *LEA* gene was found to be upregulated in wild peanut, *A*. *diogoi* upon infection with the late leaf spot pathogen, *P*. *personata* in a study of differential gene expression using cDNA-AFLP, in the previous study from same group [44]. In the present study, this partial sequence of *LEA* was amplified and the full-length cDNA sequence was obtained by 5' and 3' RACE (S1 Fig), which was further confirmed by sequencing. This sequence was designated as *AdLEA* on the basis of sequence homology it showed with LEA proteins and submitted to GenBank under NCBI accession number GU223575. *AdLEA* cDNA comprises of 1387 bp, which carried an open reading frame of 972 bp flanked by 152 bp 5ʹ untranslated region and 263 bp 3ʹ untranslated region. The ORF of *AdLEA* potentially encodes a polypeptide of 323 amino acids (S2 Fig). The theoretical *p*I and the molecular mass of AdLEA are 4.74 and 36.071 kDa respectively. AdLEA protein contains “LEA_2” motif (PF03186,1.38e-15), which was classified into subgroup 5C (D-95) according to Battaglia’s classification of LEA proteins [29].

**Fig 1:**
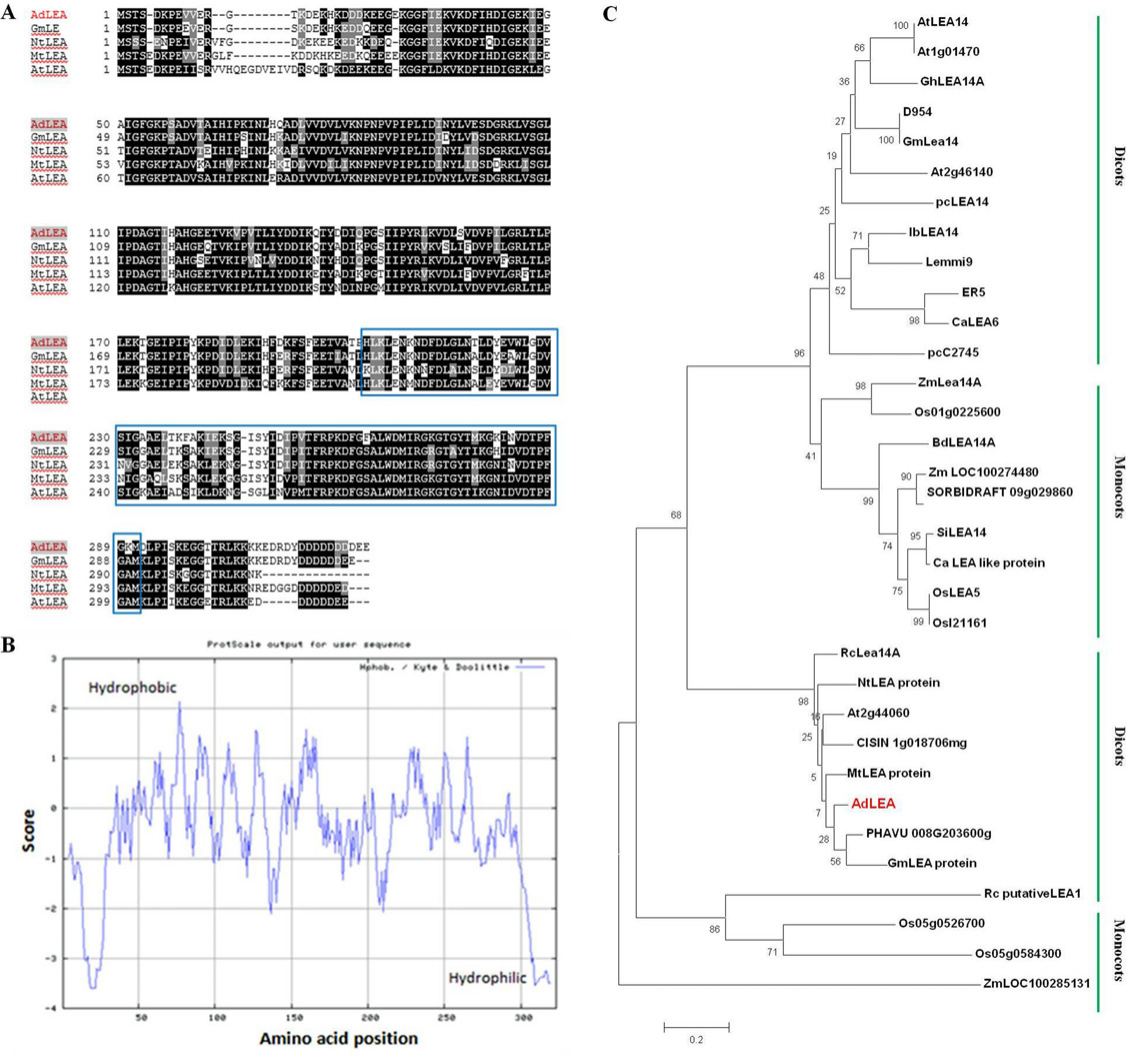
The sequence alignment, phylogenetic tree analysis and hydropathy curve of AdLEA. (A) Alignment of deduced amino acid sequence of AdLEA with other closely related LEA proteins from other plant species. The conserved “LEA_2” motif (PF03168) is boxed. AdLEA (*Arachis diogoi*, GU223575); GmLEA (*Glycine max*, KHN11020.1) NtLEA (*Nicotiana tabacum*, ABS50432.1) MtLEA (*Medicago truncatula*, XP_013459494) AtLEA (*Arabidopsis thaliana*, BT024723). (B) Hydropathy curve for AdLEA protein. (C) Phylogenetic analysis of AdLEA with LEA homologues from different plant species. The divergence of the clades between the monocots and dicots is highlighted. Bootstrap values are indicated at the branches. The accession numbers of sequences used for construction of phylogenetic tree are as follows: SiLEA14 (*Setaria italica*, KJ767551); AtLEA14 (*Arabidopsis thaliana*, NM_100029); Lea14-A (*Zea mays*, NM_001159174); D95-4 (*Glycine max*, U08108); IbLEA14 (*Ipomoea batatas*, GU369820); ER5 (*Solanum lycopersicum*, U77719); Lemmi9 (Solanum lycopersicum, Z46654); CaLEA6 (*Capsicum annuum*, AF168168); OsLEA5 (*Oryza sativa*, JF776156); pcC27-45 (*Craterostigma plantagineum*, M62990)); pcLEA14 (*Pyrus communis*, AF386513); At1g01470 (*Arabidopsis thaliana*, BT015111); LEA14-A (*Gossypium hirsutum*, M88322); Lea14 homolog (*Glycine max*, NM_001251780); At2g46140 (*Arabidopsis thaliana*, NM_130176); Os01g0225600 (*Oryza sativa*, NM_001048996); LEA14-A-like (*Brachypodium distachyon*, XM_003567779); LOC100274480 (*Zea mays*, NM_001148839); SORBIDRAFT 09g029860 (*Sorghum bicolour*, XM_002441543); LEA-like protein (*Cenchrus americanus*, AY823547); OsI21161 (*Oryza sativa*, CM000130); Os05g0526700 (*Oryza sativa*, NM_001062639); Os05g0584300 (*Oryza sativa*, NM_001062985); At2g44060 (*Arabidopsis thaliana*, BT024723); LOC100285131 (*Zea mays*, EU970969); CISIN_1g018706mg (*Citrus sinensis*, KDO49745); Th LEA protein (*Tamarix hispida*, AHF21584.1); PHAVU_008G203600g (*Phaseolus vulgaris*, XP_007141526); Lea14-A (*Ricinus communis*, XP_002533345); GmLEA (*Glycine max*, KHN11020.1) Mt LEA protein (*Medicago truncatula*, XP_013459494); chilling-responsive protein (*Nicotiana tabacum*, ABS50432); putative LEA-1 protein (*Rosa chinensis*, AKC88473).

AdLEA does not contain any signal peptide as predicted by signal P4.1 software analysis. Amino acid composition of AdLEA protein showed that it has an abundance of Asp, Lys, Ile, Gly, Leu, and Glu amino acids which constituted 12.6, 10.8, 9.5, 8.3, 7.7 and 7.6% of the total amino acid pool, respectively. Also, it lacks Cys and has just two Trp residues. Hydropathy analysis of AdLEA showed that it is a nonpolar, hydrophobic -neutral protein with a negative GRAVY value of 0.496 (Fig 1B) and has an instability index of 21.45. All these features are in accordance with other group 5C members of LEA proteins [34]. The sequence alignment of deduced amino acids of *AdLEA* was carried out with amino acid sequences of LEA proteins of other species. The alignment results by BLAST analysis showed that AdLEA protein shows the maximum similarity of 89% with LEA protein from *Glycine max*, which belongs to the D95 family of LEA proteins. It showed 79% similarity with NtLEA, 80% similarity with MtLEA and 74% similarity with AtLEA, which are LEA proteins from *Nicotiana tabacum*, *Medicago trancatula* and *Arabidopsis thaliana* respectively. The LEA_2 conserved domain was found in position about 204–299 amino acid of all these proteins (Fig 1A). Phylogenetic analysis of AdLEA showed that it is closely related to *Phaseolus vulgaris* and *Glycine max* LEA proteins, both comprising 320 amino acids as against 323 amino acids sequence of AdLEA (Fig 1C). This close similarity might be due to the fact that all three species belong to family Fabaceae.

### Relative Gene Expression Studies of *AdLEA* in Response to Phytohormones and Various Stress Treatments in *A*. *diogoi*

**Fig 2:**
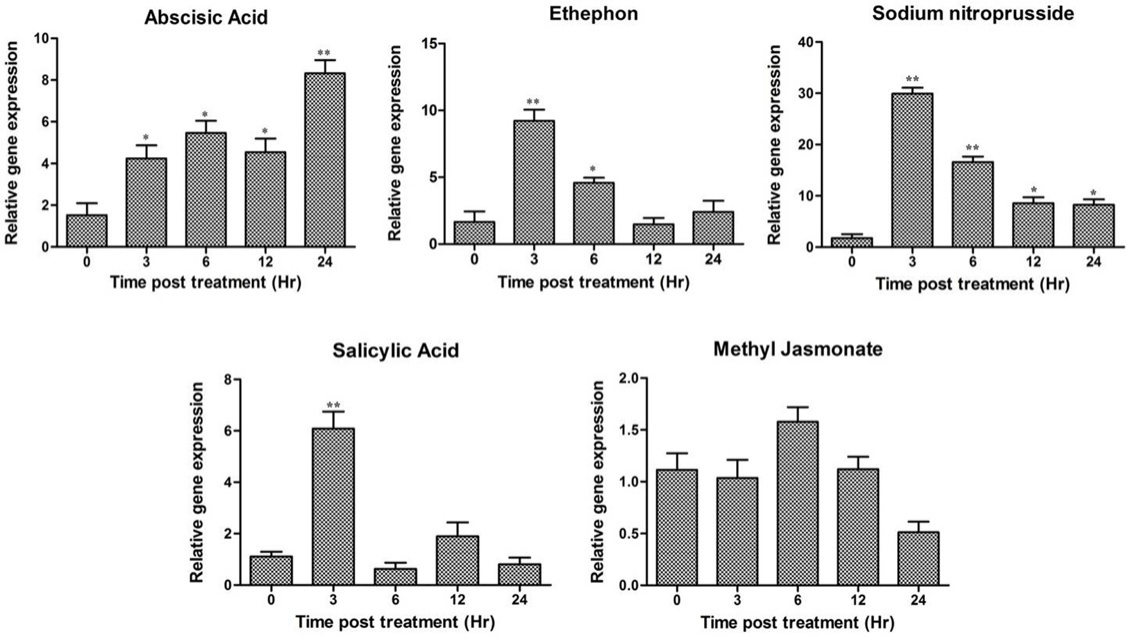
Expression profile of *AdLEA* gene in response to various phytohormones in *A*. *diogoi* using qRT-PCR. *A*. *diogoi* leaf samples were subjected to treatment with abscisic acid, salicylic acid, ethephon, sodium nitroprusside and methyl jasmonate. The samples were collected at various time intervals (in hours) and qRT-PCR was performed. Data plotted are the mean values ± SD from three independent experiments (n = 3; biological replicates). RNA from two trifoliate leaves of *A*. *diogoi* represents one biological sample. Statistical analysis was performed with one-way ANOVA (*P<0.05, **P<0.001).

**Fig 3:**
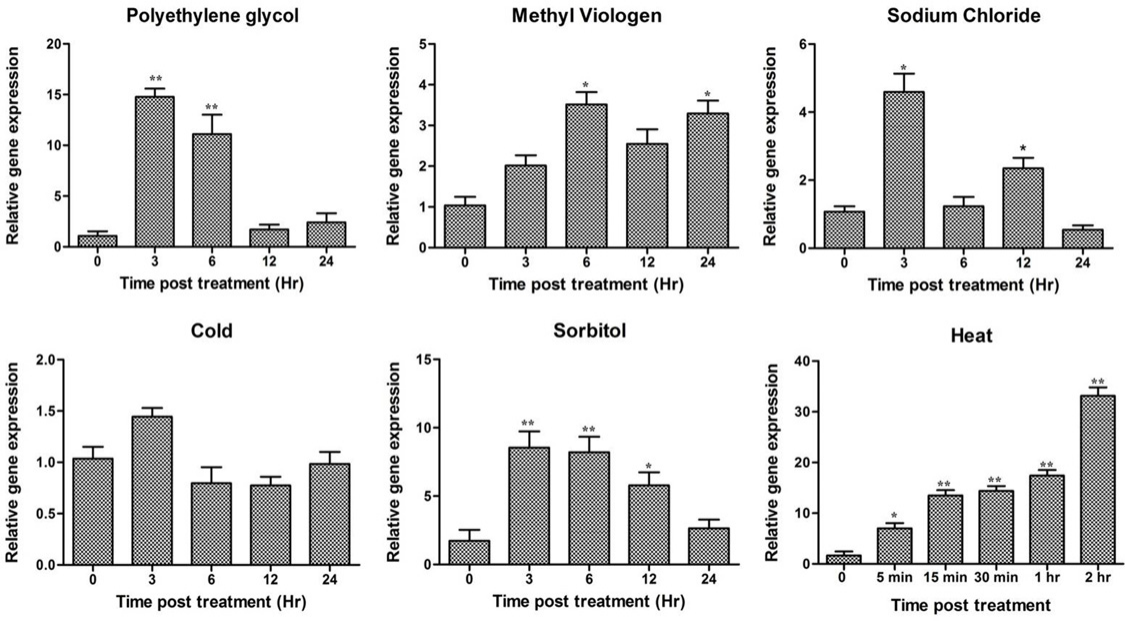
Relative gene expression of *AdLEA* in *A*. *diogoi* in response to various abiotic stresses. *A*. *diogoi* samples were treated with extreme temperatures (high and low), polyethylene glycol, sorbitol, sodium chloride and methyl viologen and *AdLEA* expression profile was analyzed by qRT-PCR. Data plotted are the mean values ± SD from three independent experiments (n = 3; biological replicates). RNA from two trifoliate leaves of *A*. *diogoi* represents one biological sample. Statistical analysis was performed with one-way ANOVA (*P<0.05, **P<0.001).

*A*. *diogoi* twigs were subjected to various treatments at different time intervals and expression analysis of *AdLEA* in response to these treatments was carried out employing qRT-PCR using the primers RTLEA-F and RTLEA-R (Table A in S1 File). The expression pattern showed that there is a basal level expression of *AdLEA* even in the absence of any treatment (depicted as 0 h in Figs 2 and 3), which gradually increased to ~8 folds after 24 h post treatment of ABA. On the other hand, there was an early 6-fold increased expression within 3 h treatment with SA followed by a gradual decrease in transcript level below basal level, 24 h post treatment. Similar patterns were observed with ethephon and SNP where there was an increase in ~10 fold and ~30-fold expression within 3 h post treatment respectively. Then, gradually the expression decreased to basal levels. There was no significant effect of meJA on *AdLEA* expression levels (Fig 2).

The highest expression of *AdLEA* was observed in high-temperature stress treatment where the expression was recorded to be beyond 30 fold within 2 h post treatment. Similarly, significant early accumulation of transcripts up to 15 fold and 8 fold were recorded for dehydration stress with PEG and osmotic stress with sorbitol, respectively within 3 h post treatment which decreased gradually. Also, there was a nearly 4-fold increase in transcript levels within 3 h post treatment with NaCl, which decreased and reached basal level after 24 h treatment. However, there was a gradual increase in expression levels from 2 to 4 fold with MV treatment in 24 h post treatment. There was no significant upregulation of *AdLEA* under low-temperature stress treatment (Fig 3).

### Sub-Cellular Localization of AdLEA

**Fig 4:**
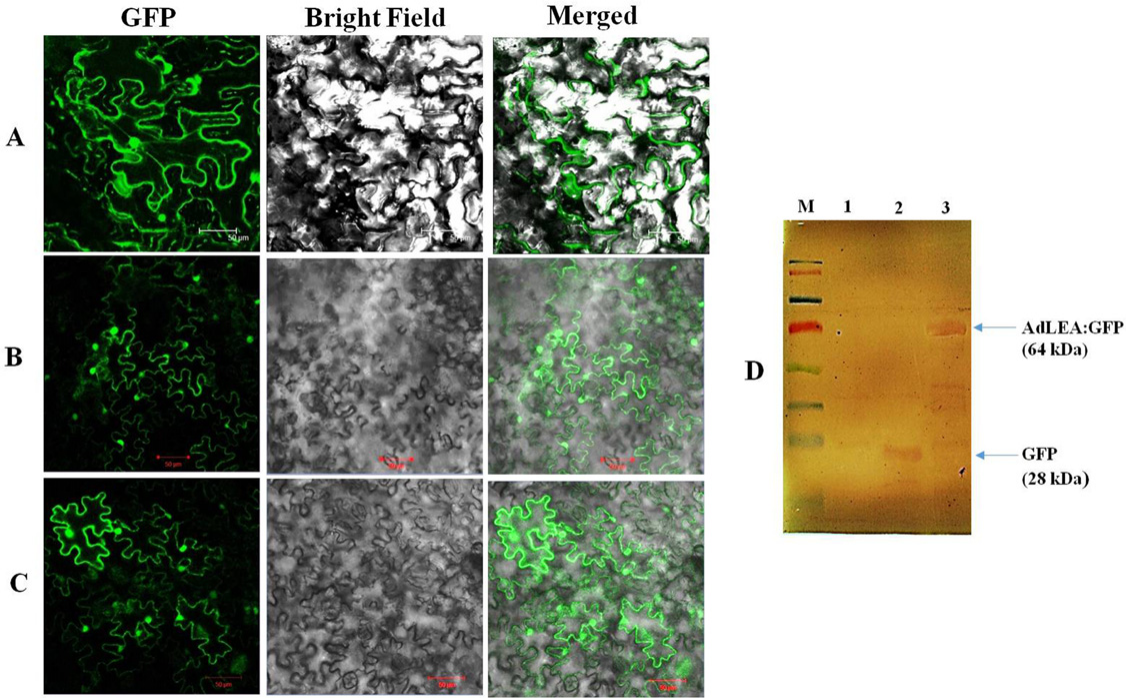
Subcellular localization of AdLEA protein in tobacco leaves. Empty pCAMBIA1302 vector serving as control and pCAMBIA1302-AdLEA and pEGAD-AdLEA recombinant vectors were transiently expressed in *N*. *benthamiana* leaves through agroinfiltration and GFP fusion proteins localization was visualized through confocal laser scanning microscope. (A) Control pCAMBIA1302 vector showing GFP throughout the cell. (B) pCAMBIA1302-AdLEA recombinant vector showing GFP in nucleus and cytosol. (C) pEGAD- AdLEA recombinant vector showing GFP in nucleus and cytosol (Bar 50 μm). Experiment was repeated three times and the representative pictures of best result among three is shown in Figure. (D) Western blot analysis of recombinant pCAMBIA1302-AdLEA protein in agro infiltrated area of leaves. Samples were collected at 72 h post agroinfiltration. M- represents pre-stained protein marker, 1- represents negative control, 2- represents a sample from positive control pCAMBIA1302 vector containing GFP-tagged with Histidine, 3- represents samples of plants infiltrated with AdLEA fused with GFP.

Subcellular localization predictions programs PSORTII, MultiLOC and Y LOC predict localization of AdLEA to be mainly cytosolic. N and C-terminal GFP translational fusion expression vectors of *AdLEA* were constructed under the control of 35 S promoter and transiently expressed in tobacco leaves by agroinfiltration, to assess the localization of AdLEA in plant cells. Confocal laser fluorescence microscopy results showed that both the N and C-terminal AdLEA: GFP fusion proteins were localized to the cytosol and nucleus of the cell and there was no impact of either of the fusions on the localization pattern of the fused proteins (Fig 4B and 4C). The expression of the fusion protein was subsequently confirmed by Western Blot (Fig 4D) which also showed that the AdLEA: GFP fusion protein was not degraded and intact in the cells.

### Genetic Transformation of Tobacco and Molecular Analysis of Transgenic *AdLEA* Tobacco Plants

*Agrobacterium* strain *EHA105* harboring the pCAMBIA2300-*AdLEA* construct under the control of CaMV35S promoter was used for transformation of *N*. *tabacum* var. Samsun. The putative T_0_ transgenic plants were screened by PCR amplification for *AdLEA* and marker *nptII* genes from the genomic DNA of plants. Out of 10 plants initially selected on kanamycin, 9 plants showed PCR amplification of 972 bp ORF of *AdLEA* and 739 bp of the *nptII* gene (S3 Fig). These plants were further analyzed by semi-quantitative RT-PCR to assess the relative expression of *AdLEA*. which demonstrated that plants #2, 4, 5, 6 and 9 showed high expression and plants #1, 7 and 8 relatively low expressions of *AdLEA* transcripts.

### Plant Morphology, PS(II) Efficiency and Proline and Chlorophyll Content in *AdLEA* Transgenic under Progressive Drought Stress and Recovery

**Fig 5:**
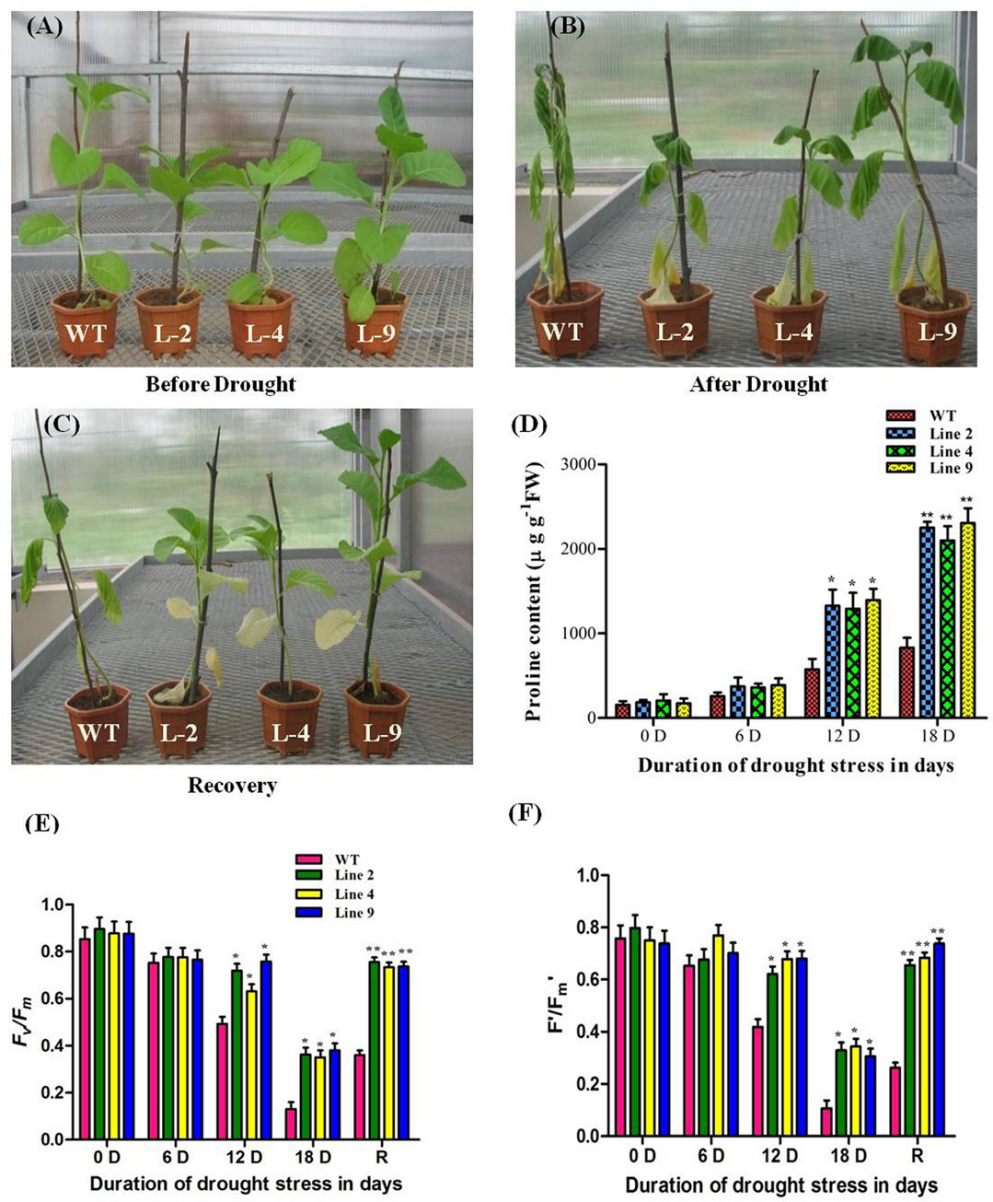
Morphological and physiological variation in WT and *AdLEA* transgenic plants under progressive drought stress. (A) Plants before drought induction; (B) during severe drought; and (C) recovery phase after drought. (D) Estimation of proline content in leaves of WT and transgenic plants during drought stress. (E and F) Chlorophyll fluorescence measurements of plants during drought stress and recovery phase; F_v_/F_m_ and Fʹ/F_m_ʹ for both WT and transgenic plants. Data plotted are the mean values ± SD from three independent experiments (n = 3; biological replicates). Single leaf from each plant constitute one biological sample. Statistical analysis was performed with two-way ANOVA (*P<0.05, **P<0.001).

The *AdLEA* expressing tobacco transgenic demonstrated enhanced tolerance under progressive drought stress as evident from the morphological differences recorded during drought and recovery in comparison to WT plants (Fig 5A, 5B and 5C). The WT leaves exhibited extensive wilting and bleached-out appearance under progressive drought stress for 18 days in comparison to leaves of transgenic plants, which were less affected. Interestingly, even after withdrawal of drought treatment for 3 d, the leaves of WT plants displayed wilting symptoms, whereas transgenic plants recovered completely and leaves appeared healthy (Fig 5C). There was no significant difference in leaf proline content of WT and transgenic plants at the initiation of the treatment (D0). As the duration of stress increased, variations in proline content were recorded between WT and transgenic plants. These differences were more evident on the 12^th^ and 18^th^ day of drought treatment with a significantly higher level of proline on the 18^th^ day in transgenic plants when compared to WT plants (Fig 5D).

Drought-induced changes in the maximal and effective photochemical quantum yield of WT and transgenic plants from chlorophyll fluorescence measurements are illustrated in (Fig 5E and 5F). There was no significant difference observed in *F*_*v*_*/F*_*m*_ on D0 and D6 between WT and transgenic plants. However, the *F*_*v*_*/F*_*m*_ of WT plants on D12 was significantly decreased by ~31% in comparison to transgenic plants, which were similar to D0 and D6. At D18 (i.e. extreme drought conditions), the *F*_*v*_*/F*_*m*_ of both WT and transgenic plants significantly decreased with the WT plants exhibiting a steep decline (~66%) in comparison to D12. The *F*_*v*_*/F*_*m*_ values significantly correlated with the chlorophyll content of the leaves of *AdLEA* transgenic plants under progressive drought stress (S4 Fig). Later, after withdrawal of drought conditions (i.e. recovery) the *F*_*v*_*/F*_*m*_ recorded for transgenic plants were similar to D0 in comparison to WT which recorded significantly lower *F*_*v*_*/F*_*m*_ similar to D12 (Fig 5E). A similar pattern was observed in *F′/F*_*m*_*′* [Y(II)] for both WT and transgenic plants demonstrating improved PS(II) efficiency under progressive drought stress and recovery (Fig 5F).

### Abiotic Stress Tolerance Assays for Transgenic *AdLEA* Plants: Dehydration and Osmotic Stress Tolerance

**Fig 6:**
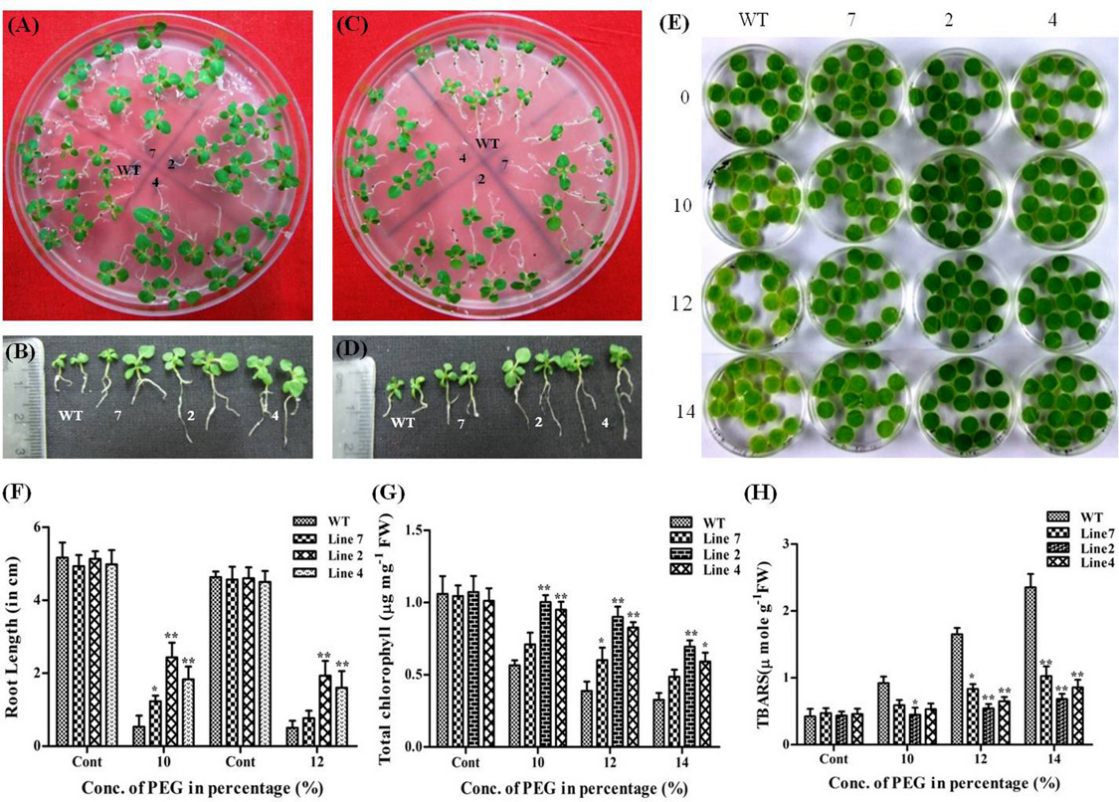
Effect of dehydration stress on *AdLEA* transgenic plants. (A and C) WT and transgenic seedlings after growth on MS medium supplemented with 10% and 12% PEG respectively. (B and D) Morphology of individual seedlings after growth on 10% and 12% PEG respectively depicting differences in root length between the seedlings. (E) Phenotypic differences between WT and transgenic leaf discs floated in dose-dependent concentrations (0, 10, 12 and 14%) of PEG. (F) Graphical representation of root length in PEG-treated seedlings after 25 d and 20 d growth on 10% and 12% PEG respectively. (G) Graphical representation showing the chlorophyll content (μg mg^-1^ FW) in the leaf discs after 72 h of treatment with 10, 12 and 14% PEG. (H) Lipid peroxidation expressed as TBARS content (μmol g^-1^ FW) in leaf discs after 72 h of treatment with 10, 12 and 14% PEG. All the experiments were performed in triplicates and data represented as mean ± SD (n = 3; biological replicates). 11 seedlings per plate constitute one biological sample for seedling assay. 15 leaf discs from a single leaf constitute one biological sample for disc senescence assay. Statistical analysis was performed with two-way ANOVA (*P<0.05, **P<0.001).

PEG and sorbitol were used for mimicking dehydration and osmotic stress conditions in transgenic plants at both seedling stage and in leaf discs from mature plants. Seedlings were allowed to grow for 25 d on 10% PEG containing media, effect of which on seedlings is depicted in (Fig 6A). No visible bleaching of seedlings was observed in WT and transgenic lines but there were prominent differences in the growth of seedlings of WT and transgenic lines. Shoots of WT seedling were stunted with reduced leaf area in comparison to transgenic seedlings. Also, there was a significant difference in the root length between WT and transgenic seedlings with the root length of transgenic plants being ~2–2.5 folds higher than that of WT (Fig 6B and 6F). The difference between WT and transgenic plants started appearing after 20 d of growth in 12% PEG medium. There was a reduction in the overall size of WT seedlings in comparison to transgenic seedlings with further reduced leaf lamina. Few seedlings showed signs of chlorosis as well (Fig 6C). The roots of transgenic seedlings were long and slender with well-developed lateral roots and the root length being ~1.5–2 folds more than that of WT seedlings (Fig 6D and 6F). However, there were no significant differences recorded in growth between seedlings of WT and low expression line, signifying the quantitative effect of degree of expression of *AdLEA* in ameliorating stress effects. Leaf discs from fully extended leaves of two-month-old plants were excised and allowed to float over 10 mL solutions of 10, 12 and 14% of PEG and distilled water (control) for 3 d under continuous white light for leaf senescence assay. During 3^rd^ day of incubation, leaf discs from WT demonstrated bleaching with increasing concentration of PEG, while there was very little bleaching observed in leaf discs from transgenic plants even in the maximum concentration of PEG, which stayed green and healthy as control discs in water (Fig 6E). The total chlorophyll content of these leaf discs after 3 d of treatment was also in accordance with the phenotype observed in them. The chlorophyll content was significantly higher in the leaf discs of the transgenic plants at all concentration of PEG when compared to WT, which showed dose-dependent loss of chlorophyll pigment. The decrease in chlorophyll content was 47% to 70% with increasing concentration of PEG in WT and 32% to 53% in low expression line when compared to water controls whereas this decrease was 6% to 41% in high expression transgenic lines (Fig 6G). The level of oxidative stress as a consequence of dehydration treatment was also determined by measuring TBARS in these discs. WT showed higher TBARS levels, the increase being 120% to 450% with increasing PEG concentration than leaf discs of all three transgenic lines which showed increments within a range of 5% to 117% with respect to control leaf discs indicating lesser lipid peroxidation and more membrane integrity under stress in transgenic plants (Fig 6H).

**Fig 7:**
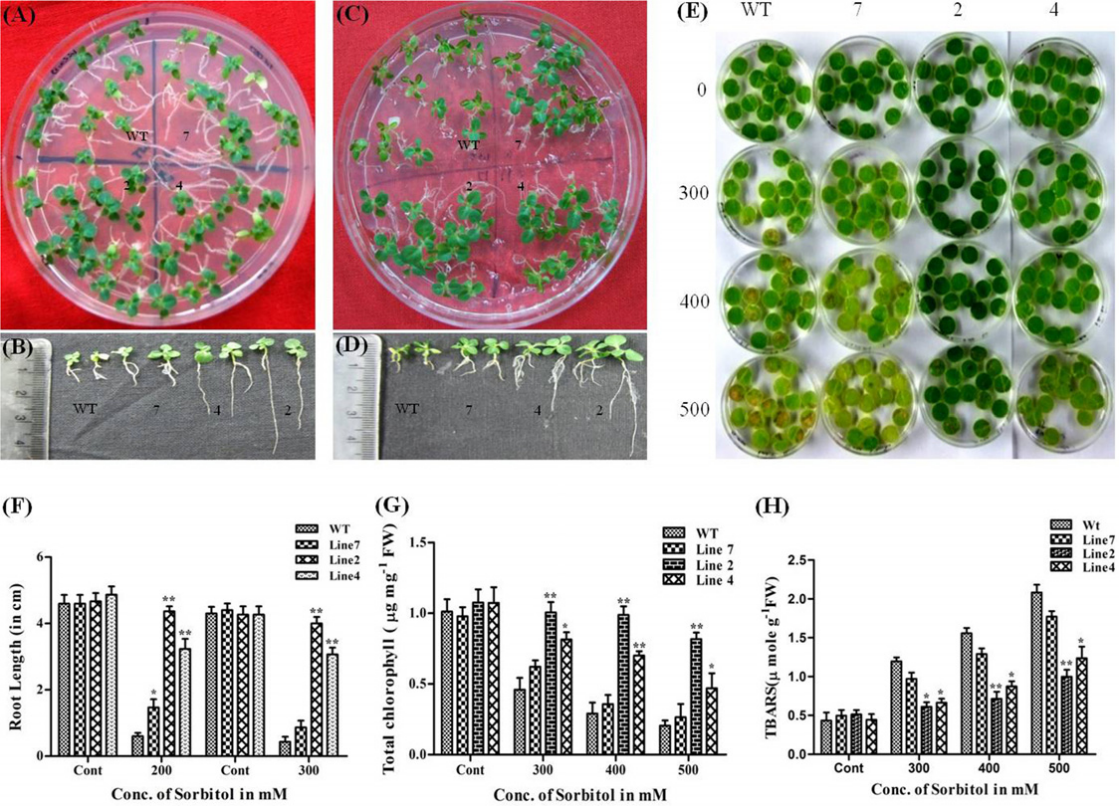
Effect of osmotic stress on *AdLEA* transgenic plants. (A and C) WT and transgenic seedlings after growth on MS medium supplemented with 200 mM and 300 mM sorbitol respectively. (B and D) Seedling morphology after growth on 200 mM and 300 mM sorbitol respectively, depicting difference in root lengths between the seedlings. (E) Phenotypic differences between the leaf discs from WT and transgenic plant lines floated in dose-dependent concentrations (200mM and 300mM) of sorbitol. (F) Graphical representation of root length in sorbitol-treated seedlings after 20d and 16d growth on 200mM and 300mM sorbitol respectively. (G) Chlorophyll content (μg mg^-1^ FW) in the leaf discs after 72h treatment with sorbitol. (H) Lipid peroxidation expressed as TBARS content (μmol g^-1^ FW) in leaf discs after 72h treatment with sorbitol. All the experiments were performed in triplicates and data represented as mean ± SD (n = 3; biological replicates). 11 seedlings per plate constitute one biological sample for seedling assay. 15 leaf discs from a single leaf constitute one biological sample for disc senescence assay. Statistical analysis was performed with two-way ANOVA (*P<0.05, **P<0.001).

Similar morphological and growth responses as in dehydration stress were recorded for transgenic plants under osmotic stress with sorbitol at two different concentrations (200 mM and 300 mM). There was significant bleaching of leaves and retarded shoot and root growth in WT seedlings in comparison to transgenic seedlings in 200 mM sorbitol (Fig 7A, 7B and 7F). Interestingly, the transgenic seedlings of high expression lines demonstrated superior growth performance even in 300 mM when compared to WT seedlings (Fig 7C, 7D and 7F). This difference was more evident in roots of transgenic and WT seedling. High expression transgenic seedlings had long slender roots with the profuse lateral root system and root length being ~3.5 to 5 folds and ~3 to 4.5 folds higher than that of WT seedlings in the case of 200 mM and 300 mM sorbitol respectively. Similarly, transgenic leaf discs demonstrated relatively green and healthy phenotype even at a higher concentration of sorbitol (500mM) in contrast to the leaf discs of WT after 3 d of incubation. This difference was more pronounced in high expression lines (Fig 7E). Chlorophyll and TBARS estimation results were also in agreement with the above-mentioned phenotypic observations with loss of chlorophyll pigment in WT ranging from 54% to 79% whereas it was 7% to 23%, 24% to 52% and 36% to 73% respectively in transgenic lines 2, 4, and 7 in comparison to discs floated on water (Fig 7G). TBARS content in leaf discs from transgenic plants was also significantly lower, which was in the range of 5% to 90% in both high expression lines and 95% to 257% in low expression line compared to the WT (175% to 380%) with increasing concentration of sorbitol (Fig 7H).

### Salinity Stress in Transgenic Plants

**Fig 8:**
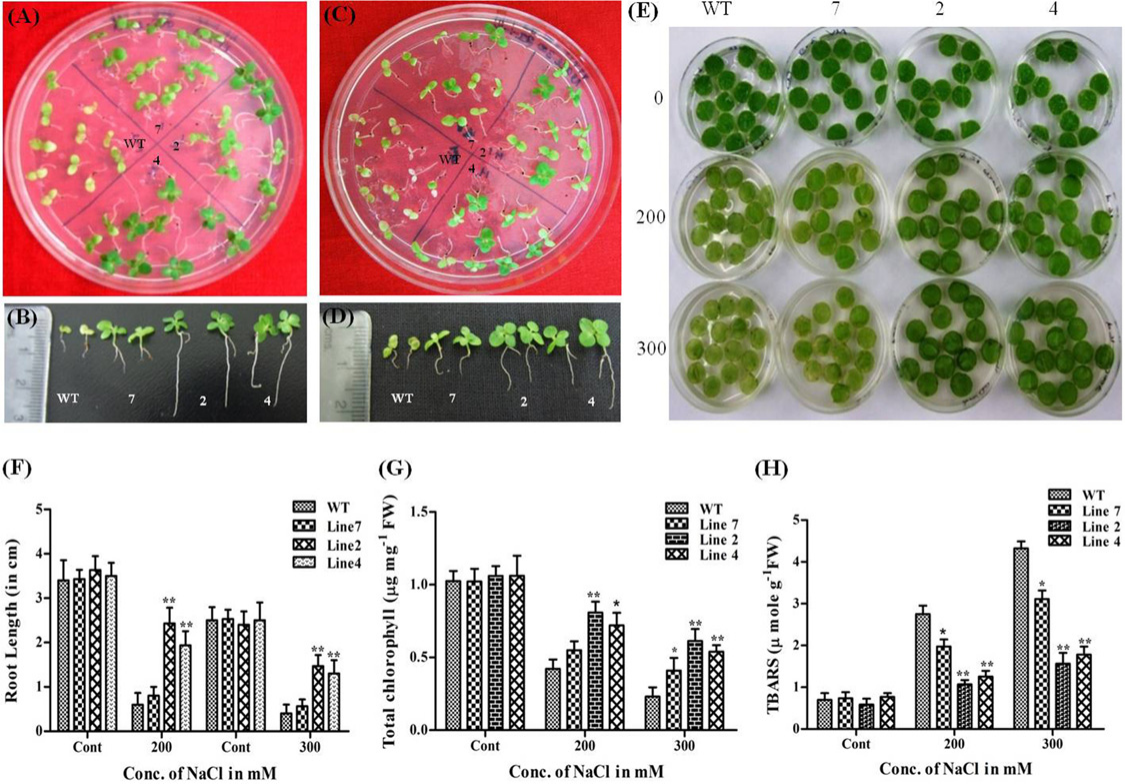
Effect of salinity stress on *AdLEA* transgenic plants. (A and C) WT and transgenic seedlings after growth on MS medium supplemented with 200mM and 300mM NaCl respectively. (B and D) Individual seedlings morphology after growth on 200mM and 300mM NaCl respectively, depicting differences in root length between the seedlings. (E) Phenotypic differences in the leaf discs from WT and transgenic plant lines floated in dose-dependent concentrations (200mM and 300mM) of NaCl. (F) Graphical representation of root lengths in NaCl-treated seedlings after 15d and 9d growth on 200mM and 300mM NaCl respectively. (G) **C**hlorophyll content (μg mg^-1^ FW) in the leaf discs after 72 h of treatment with NaCl. (H) Lipid peroxidation expressed as TBARS content (μmol g^-1^ FW) in leaf discs after 72 h of treatment with NaCl. All the experiments were performed in triplicates and data represented as mean ± SD (n = 3; biological replicates). 11 seedlings per plate constitute one biological sample for seedling assay. 15 leaf discs from a single leaf constitute one biological sample for disc senescence assay. Statistical analysis was performed with two-way ANOVA (*P<0.05, **P<0.001).

The WT seedlings showed complete growth inhibition and bleaching at 200mM NaCl after 15 d of growth whereas transgenic seedlings maintained near normal growth at this concentration with proper development of green leaves and long roots with lateral branching (root length being ~5 to 6 folds more than that of WT) (Fig 8A, 8B and 8F). This stunted growth in WT was more evident by 8^th^ day in 300mM NaCl when compared to transgenic seedlings of high expression lines which could grow normally with mild signs of chlorosis and developed healthy shoots, leaves, and roots (Fig 8C, 8D and 8F). To further investigate salinity tolerance in the growth and morphology of the mature plant, leaf discs from WT and transgenic were treated with 200mM and 300mM NaCl. Severe bleaching was observed in WT leaf discs after 2 d of treatment with 200 mM and 300 mM NaCl whereas high expression transgenic lines could withstand the stress and showed tolerance (Fig 8E) as observed from total chlorophyll content (Fig 8G) and TBARS (Fig 8H) in these lines. The low expression line showed similar response as that of WT plants (Fig 8G and 8H).

### Oxidative Stress Studies in Transgenic Plants

**Fig 9:**
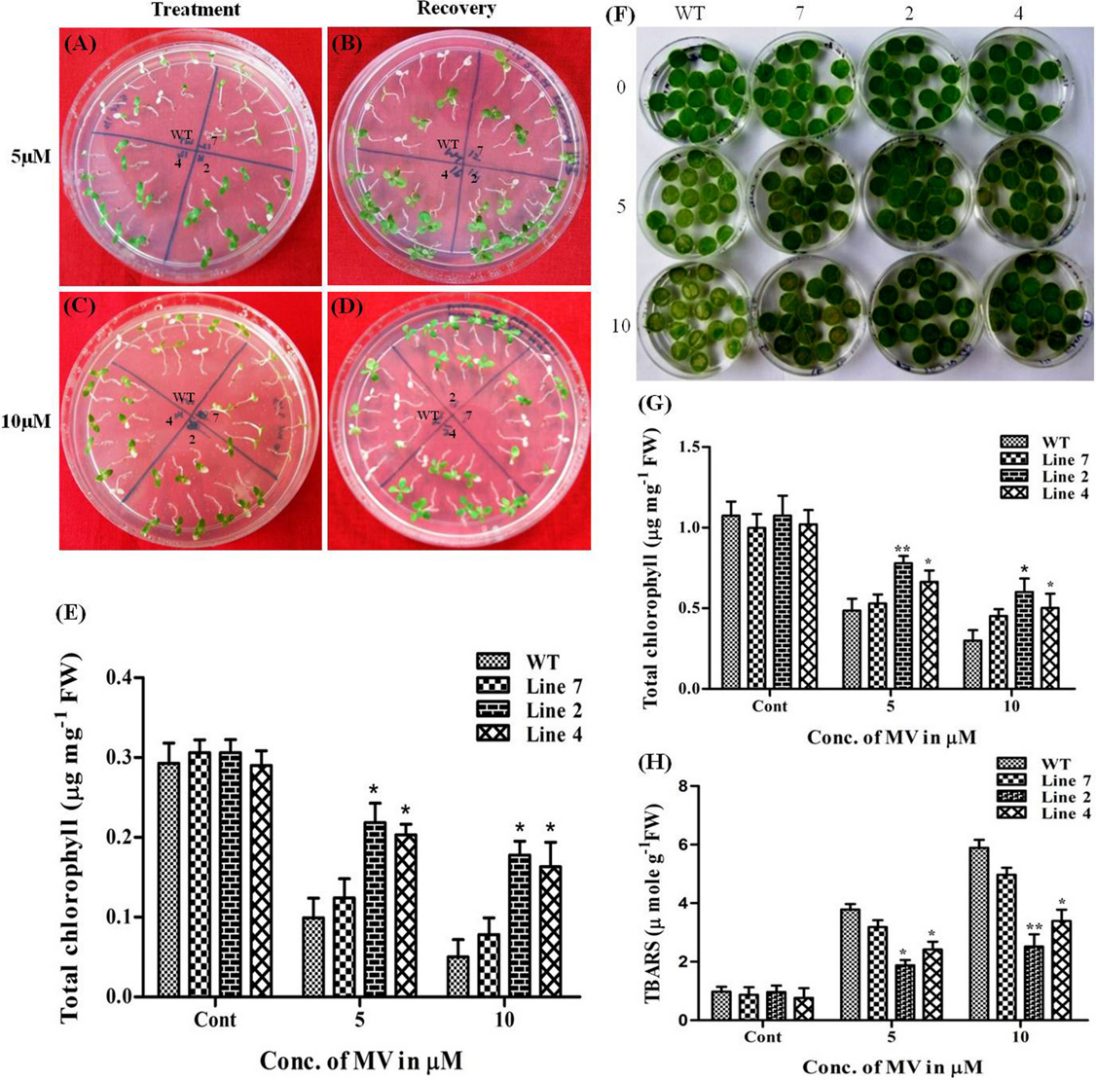
Effect of oxidative stress on *AdLEA* transgenic plants. (A and C) WT and transgenic seedlings after growth on MS medium supplemented with 5 and 10μM MV respectively. (B and D) Recovery of seedlings on MV free medium. (E) Chlorophyll content (μg mg^-1^ FW) in the seedlings after growth on MV supplemented medium. (F) Phenotypic differences in the leaf discs from WT and transgenic plant lines floated in dose-dependent concentrations (5 and 10μM) of MV. (G) Chlorophyll content (μg mg^-1^ FW) in the leaf discs after treatment with MV. (H) Lipid peroxidation expressed as TBARS content (μmol g^-1^ FW) in leaf discs after treatment with MV. All the experiments were performed in triplicates and data represented as mean ± SD (n = 3; biological replicates). 11 seedlings per plate constitute one biological sample for seedling assay. 15 leaf discs from a single leaf constitute one biological sample for disc senescence assay. Statistical analysis was performed with two-way ANOVA (*P<0.05, **P<0.001).

At 5μM MV, all the seedlings of high expression transgenic lines were healthy with negligible chlorosis whereas 70–80% of WT seedlings bleached out completely after 4 d treatment (Fig 9A). This bleached population of WT was unable to recover on MV free medium whereas seedlings of high expression transgenic lines could survive well on recovery medium (Fig 9B). Similar responses in growth as in 5μM were observed for WT and transgenic seedlings at 10μM MV (Fig 9C and 9D). The chlorophyll content in WT was recorded to be 66% to 88% less when compared to controls, which was similar to the chlorophyll content in low expression line. However, the relative decrease in chlorophyll content of high expression transgenic lines in comparison to corresponding controls was lesser and recorded between 28% to 42% and 30% to 44% respectively (Fig 9E). Leaf senescence assay performed with leaf discs also demonstrated improved performance in transgenic leaf discs in comparison to WT. Severe bleaching was observed in WT discs at both concentrations of MV with 10 μM MV being more detrimental to discs (Fig 9F). The treatment resulted in 55% to 72% loss of chlorophyll from WT as against 27% to 44% and 35% to 50% in high expression transgenic lines 2 and 4 respectively, hence recording higher chlorophyll content than WT discs (Fig 9G). TBARS content of WT was also considerably higher with a rise from 280% to 505% with increasing concentration of MV whereas this increment in TBARS was within the range of 90% to 345% in high expression transgenic lines (Fig 9H). Here also, low expression line showed a response to similar to that of WT plants with a decrease in chlorophyll and MDA content in the range of 47% to 57% and 266% to 500% respectively (Fig 9G and 9F).

### ROS Detection in Stomatal Guard Cells Using H_2_DCFDA Staining by Confocal Microscopy in *AdLEA* Transgenic Tobacco Plants

**Fig 10:**
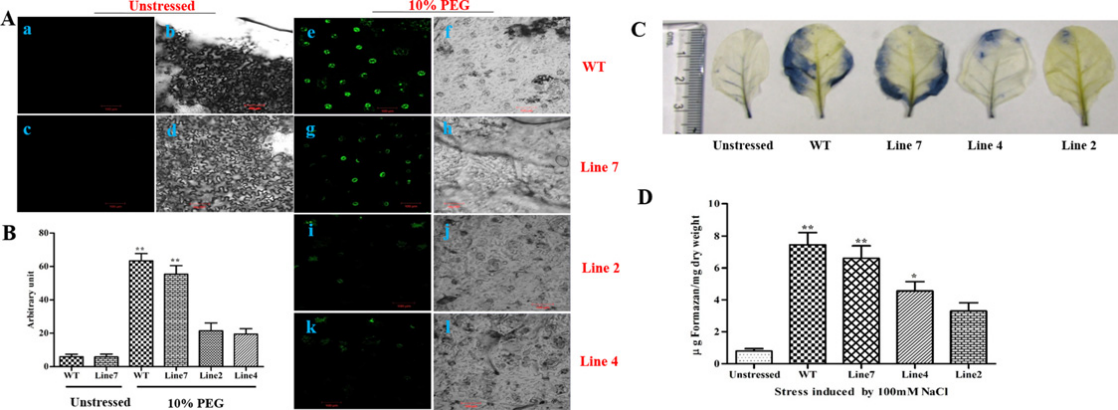
ROS detection in leaf epidermal cells by confocal microscopy and quantification of ROS in leaves using NBT staining. (A) Fluorescence levels in WT and transgenic plants before and after treatment with PEG as shown in confocal microscopy. Bright field images of WT and transgenic plants are also displayed. Stomata with water treatment. (a and b) WT; (c and d) representative transgenic line 7. Stomata after treatment with PEG. (e and f) WT; (g and h) transgenic line 7; (i and j) transgenic line 2; (k and l) transgenic line 4. The figures are representative confocal images of stomatal guard cells (n < 1000) with three biological repetitions (Bar-100μm). (B) Quantification of ROS production in cells after H_2_DCFDA staining using ImageJ software. (C) Unstressed (untreated), WT, and transgenic leaves treated with 100mM NaCl as visualized after NBT staining. (D) Graphical representation of formazan content (μg mg^-1^ dry weight) in leaves of unstressed, WT, and transgenic leaves after treatment with 100mM NaCl. All the experiments were performed in triplicates and data represented as mean ± SD (n = 3; biological replicates). Single leaf from each plant constitute one biological sample. Statistical analysis was performed with one-way ANOVA (*P<0.05, **P<0.001).

The level of DCF fluorescence under control condition (non-stress) was almost similar in all three transgenic lines and WT, thus, representative guard cell fluorescence of only one transgenic line was shown in Fig 10A (a, b, c, and d). There was an increase in florescence signal after treatment with 10% PEG in all the lines including WT; however, this increment was much intense in stomatal guard cells of WT and low expression line in comparison to the high expression transgenic lines. This indicates lower levels of ROS formation in high expression transgenic lines when compared to WT and low expression line under stress [Fig 10A (e, f, g, h, i, j, k, and l)]. There were nearly 10 folds and 8 folds’ increase in the fluorescence levels in WT and low expression line respectively under stress conditions as opposed to both high expression transgenic lines 2 and 4, where there is an increment of only 2.5 folds and 2 folds in fluorescence respectively after PEG treatment (Fig 10B).

### Detection and Quantification of ROS in Leaves Using NBT Staining

The extracts from unstressed leaf samples (serving as controls) showed negligible staining of formazan precipitate and also very little absorbance in comparison to the treated samples indicating low levels of superoxide radicles in non-treated samples. Both WT and transgenic leaves treated with 100 mM NaCl showed significantly high levels of absorbance implicating its role in inducing oxidative stress in plants. The WT showed very high levels of formazan precipitate formation as shown by intense blue coloration covering almost entire lamina in comparison to high expression transgenic lines in which only traces of blue formazan staining can be seen (Fig 10C). The levels of formazan formation in the low expression line were almost similar to WT plant. Similar results were observed when formazan levels were quantified and absorbance was measured (Fig 10D).

### Expression Analysis of Some Stress Related Genes in *AdLEA* Transgenic Plants

**Fig 11:**
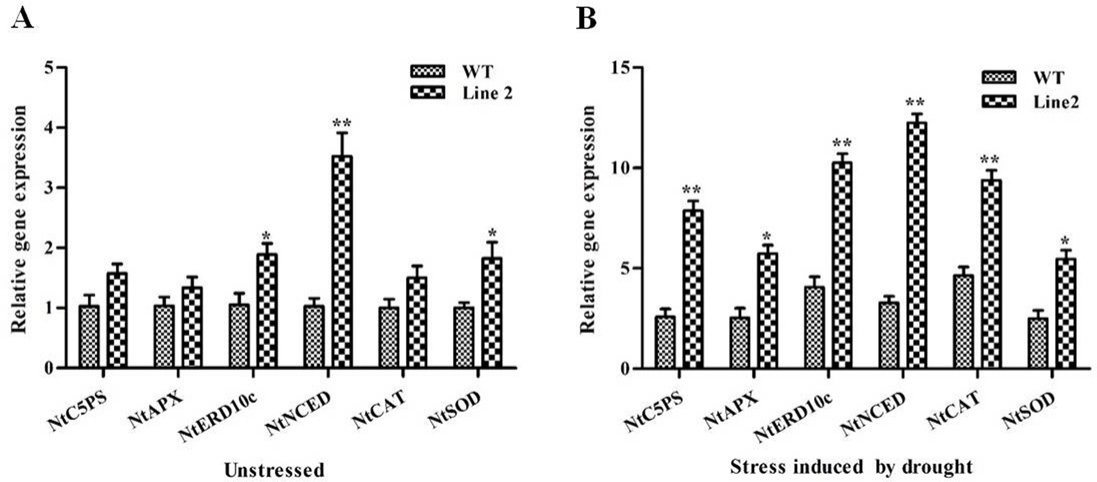
Expression analysis of stress responsive genes in *AdLEA* transgenic plants. Relative transcript levels of few abiotic stress responsive genes (*NtP5CS*, *NtAPX*, *NtERD10c*, *NtNCED*, *NtCAT*, and *NtSOD*) in *AdLEA* transgenic line #2 compared with WT were analyzed by qRT-PCR in (A) untreated and (B) drought stress conditions. All the experiments were performed in triplicates and data represented as mean ± SD (n = 3; biological replicates). RNA from single leaf from each plant constitute one biological sample. Statistical analysis was performed with two-way ANOVA (*P<0.05, **P<0.001). The primer sequences used in this study are given in Table B in S1 File.

qRT-PCR was done for some stress-responsive genes in leaves of a representative high expression line #2 in comparison to WT to elucidate their expression before and after drought treatment. There was a ~0.5-fold basal level expression in *NtP5CS* (1-pyrroline-5-carboxylate synthetase) of *AdLEA* transgenic and WT under control conditions. However, the expression of *NtP5CS* was up-regulated under stressed conditions in both WT and transgenic plants. Its expression was ~3 fold higher in transgenic plants in comparison to WT after exposure to drought (Fig 11A and 11B). There was a trivial change in expression levels of *NtAPX* (ascorbate peroxidase), *NtCAT* (Catalase), and *NtSOD* (Superoxide dismutase) (genes for corresponding ROS scavenging enzymes) from leaves of transgenic and WT plants in the absence of stress. However, there was a significant up-regulation of all three enzyme genes, after drought stress in transgenic leaves with maximum being in *NtSOD* (2.2 folds) followed by *APX* (2.1 folds) and *NtCAT* (1.9 folds) compared to WT expression levels (Fig 11A and 11B). There was significant up-regulation (2.5 folds) in the expression of *NtNCED* (9-cis-epoxycarotenoid dioxygenase), in transgenic line compared to WT even in control conditions. After drought exposure, the transcript level of *NtNCED* increased manifold in transgenic as well as in WT with ~ 4.2 folds higher in transgenic line (Fig 11A and 11B). *NtERD10c* (early response to dehydration), encoding a group 2 LEA protein was similarly up-regulated under stress conditions. The transcript levels increased to ~2.5 folds in transgenic plants than WT plants (Fig 11A and 11B). The result of this study suggested that there were enhanced transcript levels of these abiotic stress responsive genes associated with the expression of *AdLEA* in the transgenic line in response to drought stress.

## Discussion

LEA proteins are a large family of hydrophilic proteins which have been demonstrated to be associated with tolerance against multiple stresses. LEA family has been classified into seven groups, among which members of group 1, 2 and 3 are considered typical LEA proteins, which have been studied extensively in relation to abiotic stress tolerance in plants. However, “atypical” Group 5 LEA proteins have not been studied well enough and there are fewer studies to characterize their functions related to abiotic stress tolerance mechanism. Here we have reported the functional characterization of an atypical, novel *LEA* gene from *Arachis diogoi*, which was isolated earlier as a transcript derived fragment (TDF) in a differential gene expression analysis in *A*. *diogoi* challenged with late leaf spot pathogen, *P*. *personata* [44].

In the present study, the full-length cDNA sequence was amplified from this partial fragment and the gene was designated as *AdLEA* (S1 and S2 Figs). On the basis of sequence similarity, AdLEA protein was grouped under group 5C LEA proteins and it showed maximum homology with *Glycine max* LEA protein, which belongs to D95 family of LEA proteins as well (Fig 1A and 1C). In contrast to other LEA proteins, which are very hydrophilic, D95 LEA proteins display a hydropathic character throughout the length of the protein [73]. This is evident in AdLEA as well, which is a nonpolar and neutral protein with a GRAVY value of –0.496 (Fig 1B). In plants ABA is a key phytohormone involved in plant development and responses to various biotic and abiotic stresses [74]. There is an increase in endogenous ABA levels in plant cells in response to various abiotic stresses leading to the expression of stress responsive genes [75];[76]. The stress tolerance mechanisms in the plant can be ABA-dependent or independent and osmotic stress-regulated genes can be activated through both pathways [77]; [78]. However, there was a report which suggests that for activation of LEA-like proteins, stress signaling pathways independent of ABA might not exist [79]. It has been also reported that promoter region of the group 5 LEA genes generally contain an ABA-responsive element (ABRE), which drives the induction of these genes in response to various abiotic conditions [80]. There are several reports on LEA protein expression induced by various abiotic stresses emphasizing their role in imparting abiotic stress tolerance [25];[81];[82]. Consistent with the previous reports, *AdLEA* expression was also induced by ABA, high temperature, dehydration, salinity and oxidative stresses in *A*. *diogoi* (Figs 2 and 3) indicating the potential role of *AdLEA* in response to these stresses. *AdLEA* transcripts were also induced after treatment with SNP, ethephon and SA suggesting a possible cross-talk between these phytohormones in AdLEA signaling pathway [83] (Fig 2). Subcellular localization study of LEA proteins may be correlated with understanding different aspects of their functionality and their participation in cellular protection during various stresses. Group 5 LEA proteins are located in different subcellular compartments. The *Arabidopsis thaliana* group 5 protein SAG1/AtLEA5 was localized in mitochondria [84]. The maize LEA protein Rab28 was found to be localized in the nucleoli [37]. RcLEA group 5 LEA protein from *Rosa chinensis* was reported to be localized in the cytoplasm [40]. SiLEA14 from *Foxtail millet* and JcLEA from *Jatropha curcas* were found to be localized in the nucleus and cytosol both [38];[6]. Similar localization results were observed for AdLEA protein, which was found localized in nucleus and cytosol both (Fig 4). Previous studies have demonstrated that for nuclear localization of proteins, the presence of a nuclear localization signal (NLS) and its subsequent phosphorylation are essential. The localization of the maize LEA protein RAB17 to the nucleus is dependent on its phosphorylation state [10];[85]. Similarly, the NLS-segment present in the dehydrin WCS120 plays an important role in its import in the nucleus [86]. Sequence analysis of AdLEA did not reveal any known nuclear localization signal (NLS) and it also lacks the S segment that is involved in nuclear localization [85]. Thus, it is possible that an unknown nuclear localization mechanism devoid of NLS and S segment delivers AdLEA to the nucleus [87]. Also, the majority of localization prediction programs predicts its localization to be mostly cytosolic. Hence, there is another possibility that the protein is actually cytosolic in origin but diffused passively in the nucleus [88]. Nevertheless, its localization in these places suggested a comprehensive protective role for AdLEA in plant cells which is consistent with its role of imparting enhanced tolerance against abiotic stresses.

Transgenic approaches have shown that overexpression of LEA proteins from different species in *Arabidopsi*s, Tobacco, wheat, rice, maize and many plants demonstrate better growth and morphology under abiotic stress [11]. In congruence with these reports, the *AdLEA* transgenic plants also exhibited improved tolerance towards dehydration, salinity, osmotic and oxidative stress as confirmed by the data on stress assays on seedling and leaf discs from mature plants. Many abiotic stresses induce excessive accumulation of ROS in the cell, that is detrimental to cells at high concentration leading to oxidative damage to membrane lipids, nucleic acids, and proteins. This increased damage to membranes of cell and cell organelles can, in turn, hinder the growth and development of cells [89]. Damage to chloroplasts leads to depletion in the chlorophyll levels and membrane damage leads to high MDA levels in cells [90]. The leaf disc assays showed higher chlorophyll content and less MDA levels in transgenic *AdLEA* plants in comparison to WT plants under all the stress treatments (Figs 6–9). This clearly indicated reduced lipid peroxidation and membrane damage in these plants. Similar results were observed when *in situ* H_2_O_2_ and O_2_^-^ free radicals were measured in WT and *AdLEA* transgenic plants using H_2_DCFDA fluorescence and NBT staining under dehydration and salinity stress conditions. Accumulation of ROS, specifically superoxide anion (O_2_^-^) has been used as an effective index to assess the tolerance to multiple stresses, because it directly reflects on the capability to resist oxidative stress in plants [89]. In both dehydration and salinity stresses, transgenic plants displayed enhanced antioxidative capacity in the form of decreased accumulation of O_2_—compared to WT (Fig 10). Low levels of O_2_^-^ commonly indicate higher tolerance to abiotic stresses in transgenic plants, which is consistent with the above physiological and biochemical results under different abiotic stress assays, and may also explain the enhanced tolerance to these stresses in transgenic plants.

In plants, the ability to maintain photosynthesis under environmental stress is a fundamental requirement to maintain their growth and development. The potential function of *AdLEA* in stress adaptation was further explored by monitoring the photosynthesis performance of transgenic plants. The chlorophyll a fluorescence studies demonstrated improved PS-II efficiency in *AdLEA* transgenic plants when compared to WT plants under progressive drought stress. *F*_*v*_*/F*_*m*_ reflects an estimate of the maximum quantum efficiency of PSII photochemistry and has been widely used to detect stress-induced perturbations in the photosynthetic apparatus [91]. In this study, WT plants showed a significant decrease in *F*_*v*_*/F*_*m*_ under drought stress when compared to transgenic plants suggesting the development of slowly relaxing quenching processes and photo-damage to PSII reaction centers, both of which reduce the PSII photochemistry. Similarly, *F′/F*_*m*_*′* measurement also demonstrated the same trend as for *F*_*v*_*/F*_*m*_ in WT plants and *AdLEA* transgenic plants (Fig 5). This indicates improved operational quantum efficiency of PSII electron transport and hence, CO_2_ assimilation in transgenic plants as both are directly related [92]. Even after a recovery period of 3 d after extreme drought treatment (D18), the WT plants were unable to recover on re-watering in comparison to transgenic plants as demonstrated by *F′/F*_*m*_*′* and *F*_*v*_*/F*_*m*_ measurements, suggesting irreversible damage to photosynthetic apparatus. The *AdLEA* transgenic plants showed *F′/F*_*m*_*′* and *F*_*v*_*/F*_*m*_ values similar to that of the healthy unstressed plant, which showed the ability of *AdLEA* to partially reverse the stress induced by growth inhibition under drought. The improved CO_2_ assimilation of transgenic plants under stress possibly would have allowed the plants to sustain superior growth. This better photosynthesis performance of the transgenics can also be linked to the accumulation of more proline levels and less damage to chlorophyll content in leaves of transgenic plants under drought in comparison to WT (Fig 5 and S4 Fig). Proline is an organic osmolyte, which works in multiple ways to impart stress tolerance to cells by the protection of cellular structures, detoxification of enzymes, scavenging ROS alone or in combination with other stress-related enzyme system. These compounds also confer integrity to the membrane and keep the photosynthesis intact under stress. Proline accumulation is a well-known measure adopted for the alleviation of osmotic stress in plants and it has also been documented that leaf proline content is correlated with plant tolerance to abiotic stresses [93];[94];[95].

Further, to understand the possible molecular mechanisms, which help *AdLEA* transgenic plants to combat stress in response to drought, some of the stress responsive genes were analyzed in high expression transgenic plants compared with WT. Plants possess a complex antioxidant defense system of ROS detoxification generated as a result of any induced stress, which consists of an accumulation of non-enzymatic antioxidants like proline and activation of ROS scavenging enzymes [96]. It has been reported that drought tolerance is positively correlated with the activity of antioxidant enzymes such as SOD, CAT, and APX in plants [97]. Consistent with these studies, *AdLEA* transgenic plants demonstrated enhanced expression of *NtAPX*, *NtSOD*, and *Nt*CAT in comparison to WT under drought conditions (Fig 11B), which might presumably also explain the lower levels of ROS in leaves of transgenic plants under dehydration stress induced by PEG (Fig 10A and 10B). Also, there was enhanced expression of *NtP5CS* (a key enzyme involved in the synthesis of amino acid proline in plants) under drought stress in transgenic plants in comparison to WT (Fig 11B). This result was in concordance with the data of elevated proline levels in transgenic plants during drought stress (Fig 4D). There was enhanced expression of stress responsive genes *NtERD10c* and *NtNCED* in transgenic plants when compared to WT plants (Fig 11B). *ERD10c* encodes a group 2 hydrophilic LEA protein that is assumed to play critical roles in combating cellular dehydration by binding water, stabilizing labile enzymes and protecting cellular macromolecular structures [98]. Greater induction of this gene in transgenic line suggested that plants might synthesize more protective chaperones for protein stabilization providing better defense against water loss in dehydration or drought stress as opposed to WT plants [99]. NCED is a rate limiting key enzyme in the biosynthesis of ABA, which plays an essential role in adaptive responses to environmental stresses including drought stress [100]. Interestingly, there was enhanced expression of *NtNCED* in transgenic plants in comparison to WT plants in control conditions before stress treatment, which became more pronounced after drought stress (Fig 11A and 11B). This suggests increased *de novo* ABA biosynthesis in transgenic plants, which could have been promoted by the constitutive expression of *AdLEA* in these plants. The assumption sounds reasonable since there are reports where an increase in the endogenous ABA level by expression of *NCED* gene resulted in improved drought tolerance in various plants [101]. This study on these genes (*NtERD10*, and *NtNCED*) reconfirm the fact that their associated enhanced expression in *AdLEA* transgenic plants appeared to facilitate plant tolerance towards drought stress. Hence, the enhanced tolerance of *AdLEA* transgenic plants against dehydration or drought stress could be attributed to the cumulative effects of up regulation of many genes and effects of their gene products. *AdLEA* transgenic plants demonstrated higher antioxidant activity owing to enhanced expression of antioxidant enzymes under stressed conditions, which might have aided the transgenic plants to scavenge ROS more effectively than WT plants. Similarly, up-regulation of stress-responsive genes involved in proline metabolism and ABA signaling pathways might have played a role in conferring enhanced tolerance to drought stress in *AdLEA* transgenic plants. Upregulation of an LEA homolog of tobacco (*ERD10*) and its functions in imparting stress tolerance can also be associated with increased tolerance of *AdLEA* transgenic plants.

To summarize this study characterizes a novel, atypical group 5 *LEA* gene from *A*. *diogoi*. *AdLEA* is responsive to ABA, PEG, NaCl, sorbitol, high temperature and MV and is localized in cytosol and nucleus. Overexpression of *AdLEA* imparts abiotic stress tolerance to transgenic tobacco plants most specifically in water limiting conditions by increasing O_2_^-^ scavenging and up-regulation of various stress-related genes. *AdLEA* can be a good candidate gene in crop improvement management program for imparting enhanced tolerance to plants towards multiple stresses.

## Supporting Information

S1 Fig RACE-PCR and cloning of *AdLEA* from *Arachis diogoi*. Representative pictures of 5ʹ and 3ʹ RACE PCR products of *AdLEA* and its Open Reading Frame (ORF).
(TIF)

S2 Fig The nucleotide sequence, deduced protein and gene structure of *AdLEA*. (A) Nucleotides are numbered and the start and stop codons are underlined and in bold. (B) The nucleotide length composition of the cDNA is shown; closed box represents the Open Reading Frame (ORF) of *AdLEA* and lines represent 5ʹ and 3ʹ UTR.
(TIF)

S3 Fig Integration and expression of *AdLEA* in transgenic tobacco plants. (A) PCR analysis of putative T_0_ transformants for the *nptII* gene; M- λDNA/*Eco*RI+*Hin*dIII ladder, 2- WT negative control, 1 and 3–10 transgenic plants showing 739 bp amplified PCR product of the *nptII* gene. (B) PCR analysis of putative T_0_ transformants for *AdLEA* gene; M- Marker, 1- negative control (without DNA), 2- positive control vector, 3- WT negative control, 4 to12 transgenic plants showing 972 bp amplified PCR product of *AdLEA* gene. (C and D) Transcript levels of *AdLEA* in T_0_ and T_2_ generation. Line 2, 4, 5, 6 and 9 are high expression lines and 1, 7 and 8 are low expression lines.
(TIF)

S4 Fig Chlorophyll estimation in WT and *AdLEA* transgenic plants in progressive drought stress. Graphical representation showing the chlorophyll content (μg mg^-1^ FW) in the WT and *AdLEA* transgenics plants in drought stress. Data plotted are the mean values ± SD from three independent experiments (n = 3; biological replicates). Single leaf from each plant constitute one biological sample. Statistical analysis was performed with two-way ANOVA (*P<0.05, **P<0.001).
(TIF)

S1 File Oligo and their sequences used for RACE-PCR, *AdLEA* gene cloning and integration and drought stress tolerance studies in *AdLEA* transgenics plants. (DOC)
