# Supplementary figures and images for: Ectopic Expression of an Atypical Hydrophobic Group 5 LEA Protein from Wild Peanut, Arachis diogoi Confers Abiotic Stress Tolerance in Tobacco
Source: PLoS One. 2016 Mar 3;11(3):e0150609. doi: 10.1371/journal.pone.0150609 (PMC4777422; doi:10.1371/journal.pone.0150609)

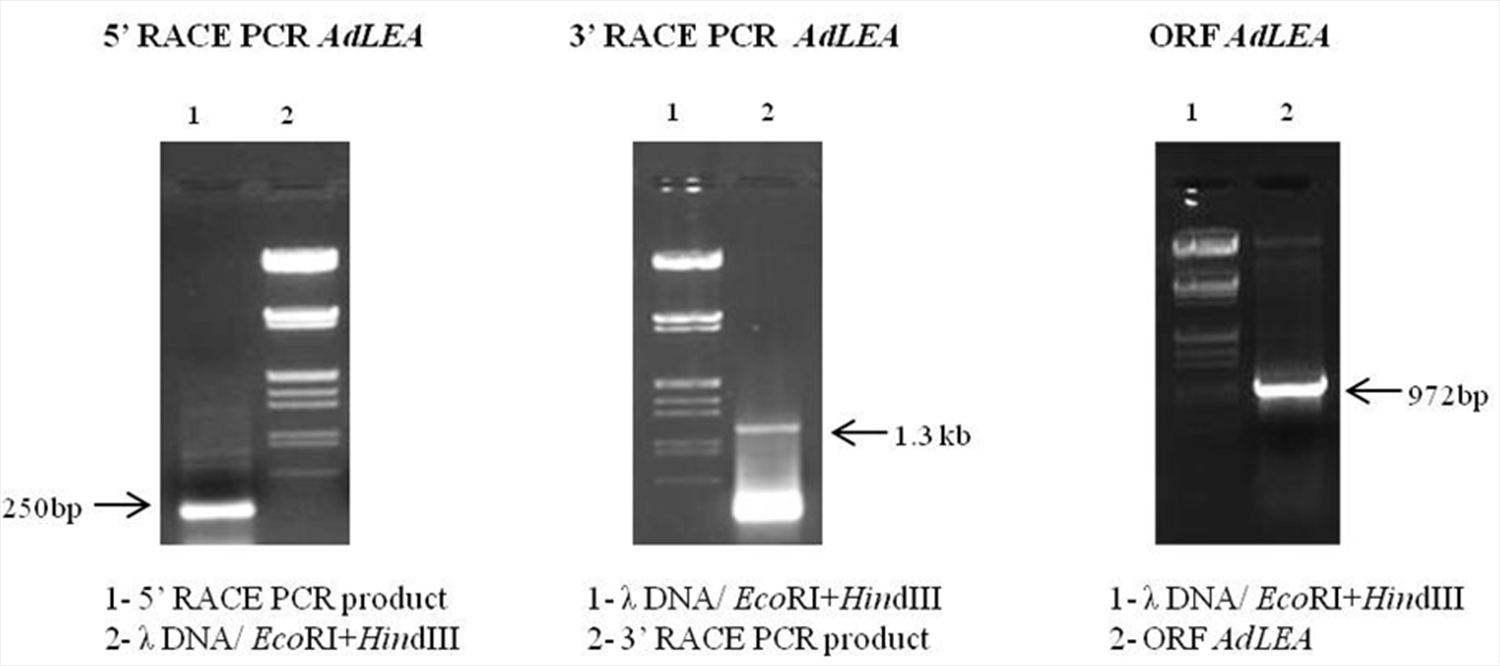

Supplement: S1 Fig — Representative pictures of 5ʹ and 3ʹ RACE PCR products of AdLEA and its Open Reading Frame (ORF). (TIF) [file pone.0150609.s001.tif]

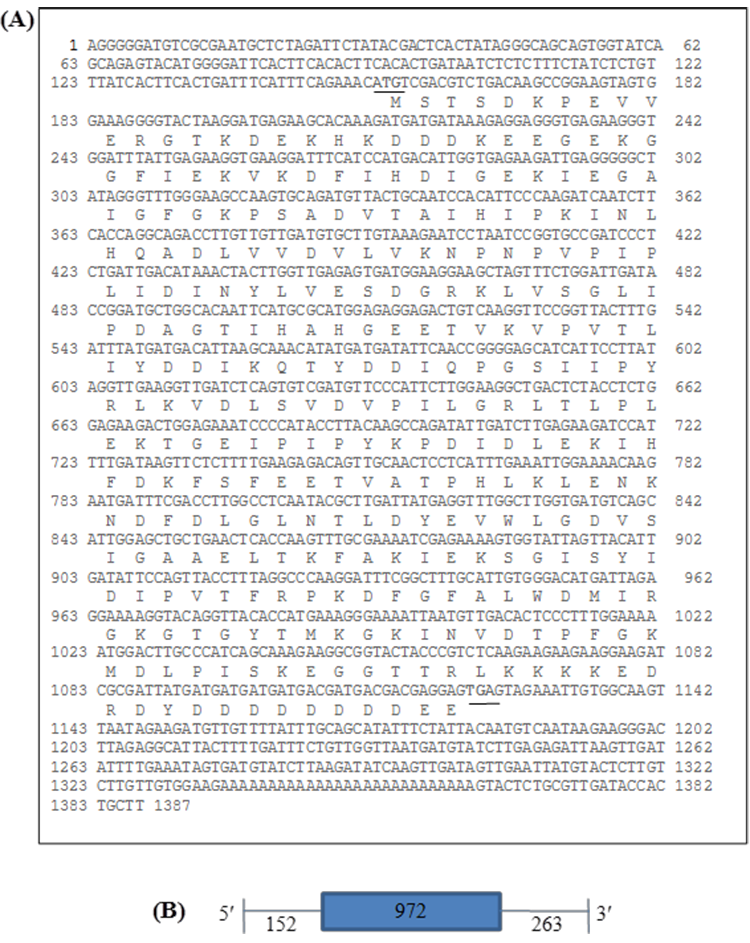

Supplement: S2 Fig — (A) Nucleotides are numbered and the start and stop codons are underlined and in bold. (B) The nucleotide length composition of the cDNA is shown; closed box represents the Open Reading Frame (ORF) of AdLEA and lines represent 5ʹ and 3ʹ UTR. (TIF) [file pone.0150609.s002.tif]

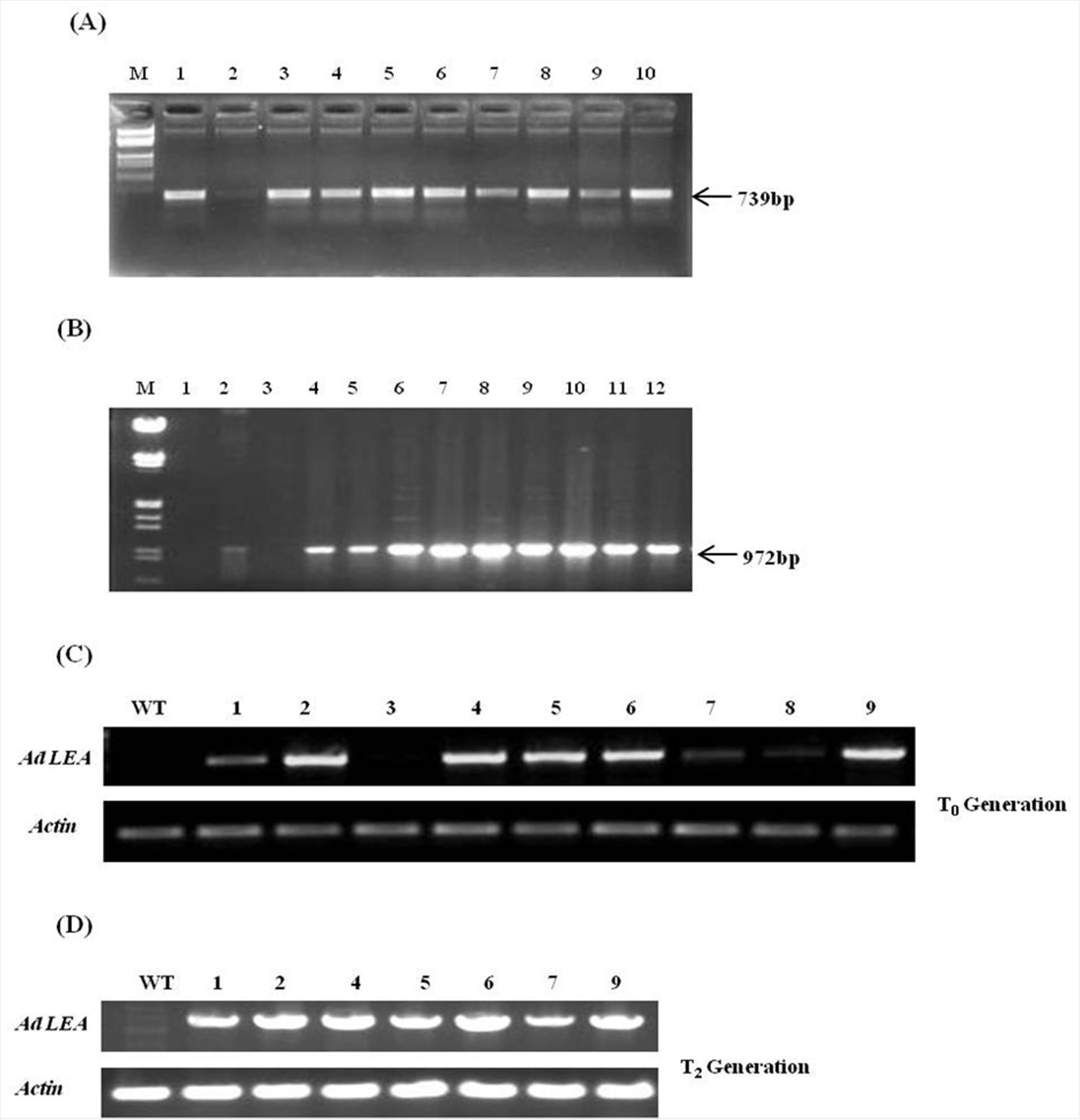

Supplement: S3 Fig — (A) PCR analysis of putative T0 transformants for the nptII gene; M- λDNA/EcoRI+HindIII ladder, 2- WT negative control, 1 and 3–10 transgenic plants showing 739 bp amplified PCR product of the nptII gene. (B) PCR analysis of putative T0 transformants for AdLEA gene; M- Marker, 1- negative control (without DNA), 2- positive control vector, 3- WT negative control, 4 to12 transgenic plants showing 972 bp amplified PCR product of AdLEA gene. (C and D) Transcript levels of AdLEA in T0 and T2 generation. Line 2, 4, 5, 6 and 9 are high expression lines and 1, 7 and 8 are low expression lines. (TIF) [file pone.0150609.s003.tif]

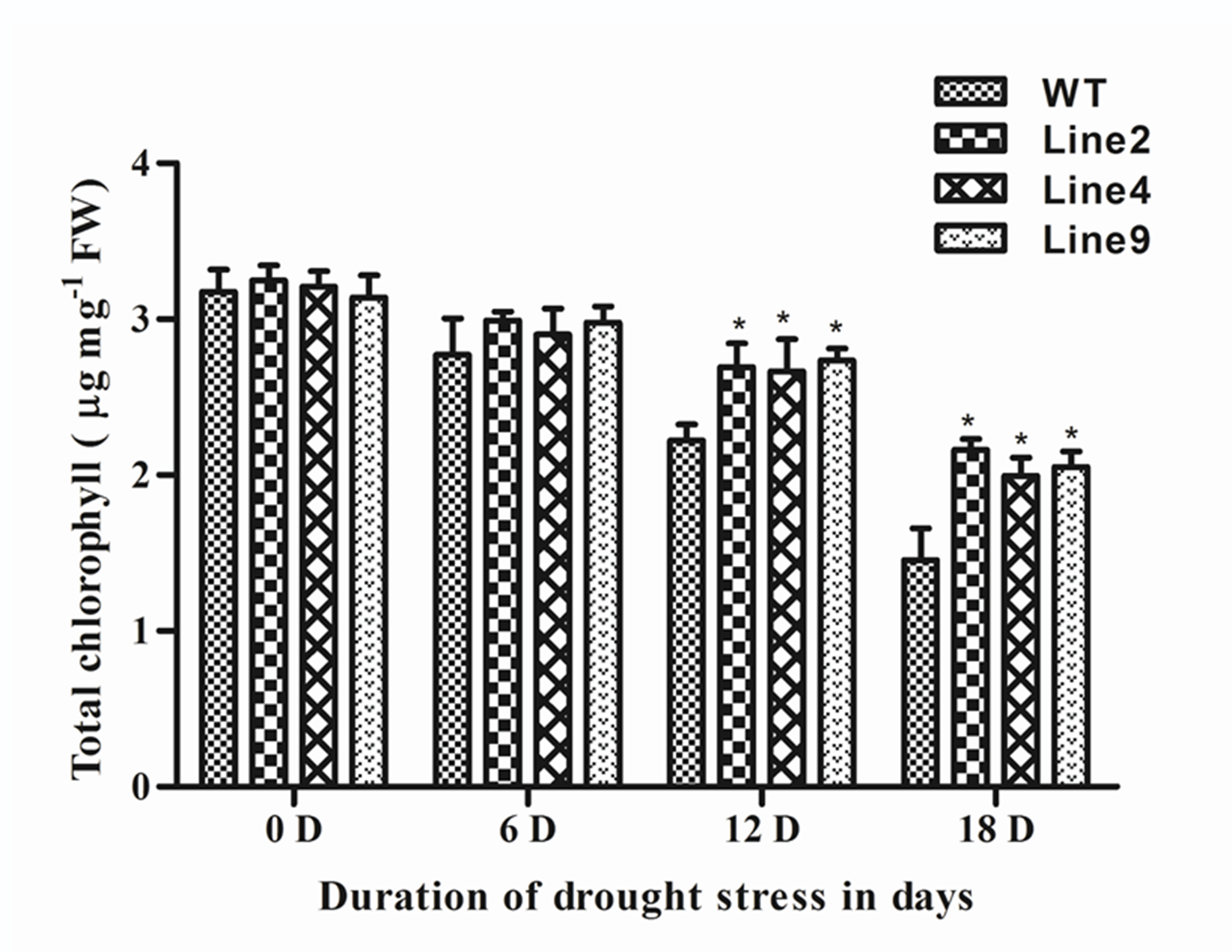

Supplement: S4 Fig — Graphical representation showing the chlorophyll content (μg mg-1 FW) in the WT and AdLEA transgenics plants in drought stress. Data plotted are the mean values ± SD from three independent experiments (n = 3; biological replicates). Single leaf from each plant constitute one biological sample. Statistical analysis was performed with two-way ANOVA (*P<0.05, **P<0.001). (TIF) [file pone.0150609.s004.tif]
