# Supplementary material for: Ectopic Expression of an Atypical Hydrophobic Group 5 LEA Protein from Wild Peanut, Arachis diogoi Confers Abiotic Stress Tolerance in Tobacco
Source: PLoS One. 2016 Mar 3;11(3):e0150609. doi: 10.1371/journal.pone.0150609 (PMC4777422; doi:10.1371/journal.pone.0150609)
Supplement: S1 File — (DOC) [file pone.0150609.s005.doc]

**Table A. Oligo and their sequences used for RACE-PCR, cloning and gene integration studies in *AdLEA*** transgenics plants.

| **Gene** | **Primer Name** | **Primer sequence (5ʹ-3ʹ)** |
| --- | --- | --- |
| *AdLEA* | 5'AdLEAGSP1 | CATCTGCACTTGGCTTCCCAAACCCT |
|  | 3'AdLEAGSP1 | AGGGTTTGGGAAGCCAAGTGCAGATG |
|  | AdLEA-F-SacI | CGAGCTCATGTCGACGTCTGACAAGC |
|  | AdLEA-R-KpnI | AGGTACCTCACTCCTCGTCGTCATCG |
|  | AdLEA-pEGAD-F | AGAATTCAT GTC GAC GTC TGA CAA GC |
|  | AdLEA-pEGAD-R | TAAGCTTTC ACT CCT CGT CGT CAT C |
|  | AdLEA-pC1302-F | CAGATCT ATGTCGACGTCTGACAAGCC |
|  | AdLEA-pC1302-R | CACTAGT CTC CTC GTC GTC ATC GTCAT |
|  | RTLEA-F | GGCTTTGCATTGTGGGACATGA |
|  | RTLEA-R | TCACTCCTCGTCGTCATCGTC |
| *nptII* | NptII-F | AGATGGATTGCACGCAGGTTCTC |
|  | NptII-R | ATCGGGAGCGGCGATACCGTA |
| *Adh3* | AdRTADH3-F | GACGCTTGGCGAGATCAACA |
|  | AdRTADH3-R | AACCGGACAACCACCACATG |
| *Actin* | Actin-F | TGGCATCACACTTTCTACAA |
|  | Actin-R | CAACGGAATCTCTCAGCTCC |
| *UBI1* | UBI1RT-F | TCTTGTCCTCCGTCTTAGGG |
|  | UBI1RT-R | AGCAAGGGTCCTTCCATCTT |
|  |  |  |
|  |  |  |

Underlined bases indicate the recognition sequences for the corresponding restriction enzymes

**Table B. Oligo sequences used in the study of drought stress tolerance in *AdLEA* transgenic tobacco** plants

| **Gene** | **Oligo Name** | **Oligo sequence (5ʹ-3ʹ)** |
| --- | --- | --- |
| *NtAPX* | NtAPX-F | GTTTGGGCTTTTCTCCTCGAC |
|  | NtAPX-R | GGAGCATAAGAGGAGCGCAA |
| *NtMnSOD* | NtMnSOD-F | TCCCCTACGACTATGGAGCA |
|  | NtMnSOD-R | CGGTATGCAATTTGGCGACG |
| *NtERD10C* | NtERD10C-F | AAAGCCAACTCATGCCCAAG |
|  | NtERD10C-R | AGAGCTGCTACTTGATCGATGG |
| *NtP5CS* | NtP5CS-F | GCTGCTCAACAGGCTGGATA |
|  | NtP5CS-R | CCATCAGCAACCTCCGTTCT |
| *NtCAT* | NtCAT-F | GGCCGCTACAACTCTCTCTTT |
|  | NtCAT-R | ACAGGACCTCTTGCACCAAC |
| *NtNCED3* | NtNCED3 F | TGTCTGAAATGATCCGGGGC |
|  | NtNCED3 R | AGTTTCCGGCTCTTCCCAAG |
| *Nt18S* | Nt18S-F | CCAGGTCCAGACATAGTAAG |
|  | Nt18S-R | GTACAAAGGGCAGGGACGTA |
| *NtUBI1* | NtUBI1- F | GAGTCAACCCGTCACCTTGT |
|  | NtUBI1- R | ACATCTTTGAGACCTCAGTAGACA |
|  |  |  |
|  |  |  |
